# The distinct initiation sites and processing activities of TTLL4 and TTLL7 in glutamylation of brain tubulin

**DOI:** 10.1016/j.jbc.2023.104923

**Published:** 2023-06-14

**Authors:** Xinyue Zhang, Xiangxiao Li, Wei Chen, Yujuan Wang, Lei Diao, Yan Gao, Heyi Wang, Lan Bao, Xin Liang, Hui-Yuan Wu

**Affiliations:** 1 School of Pharmaceutical Science and Technology, Tianjin University, Tianjin, China; 2 IDG/McGovern Institute for Brain Research, School of Life Sciences, Tsinghua University, Beijing, China; 3 State Key Laboratory of Cell Biology, Shanghai Institute of Biochemistry and Cell Biology, Center for Excellence in Molecular Cell Science, Chinese Academy of Sciences, Shanghai, China

**Keywords:** polyglutamylation, TTLL4, TTLL7, enzyme characterization, tubulin, brain development

## Abstract

Mammalian brain tubulins undergo a reversible posttranslational modification—polyglutamylation—which attaches a secondary polyglutamate chain to the primary sequence of proteins. Loss of its erasers can disrupt polyglutamylation homeostasis and cause neurodegeneration. Tubulin tyrosine ligase like 4 (TTLL4) and TTLL7 were known to modify tubulins, both with preference for the β-isoform, but differently contribute to neurodegeneration. However, differences in their biochemical properties and functions remain largely unknown. Here, using an antibody-based method, we characterized the properties of a purified recombinant TTLL4 and confirmed its sole role as an initiator, unlike TTLL7, which both initiates and elongates the side chains. Unexpectedly, TTLL4 produced stronger glutamylation immunosignals for α-isoform than β-isoform in brain tubulins. Contrarily, the recombinant TTLL7 raised comparable glutamylation immunoreactivity for two isoforms. Given the site selectivity of the glutamylation antibody, we analyzed modification sites of two enzymes. Tandem mass spectrometry analysis revealed their incompatible site selectivity on synthetic peptides mimicking carboxyl termini of α1- and β2-tubulins and a recombinant tubulin. Particularly, in the recombinant α1A-tubulin, a novel region was found glutamylated by TTLL4 and TTLL7, that again at distinct sites. These results pinpoint different site specificities between two enzymes. Moreover, TTLL7 exhibits less efficiency to elongate microtubules premodified by TTLL4, suggesting possible regulation of TTLL7 elongation activity by TTLL4-initiated sites. Finally, we showed that kinesin behaves differentially on microtubules modified by two enzymes. This study underpins the different reactivity, site selectivity, and function of TTLL4 and TTLL7 on brain tubulins and sheds light on their distinct role *in vivo*.

Polyglutamylation is a reversible protein post-translational modification, by which proteins are attached with a side chain of glutamate residues. First, the γ-carboxyl of a glutamate residue in the primary structure of protein is linked to a free glutamate through an amide bond to form the branch point. Variable number of glutamate residues are then sequentially added mainly through α-carboxyl linkage to form a side chain (1). Members of the tubulin tyrosine ligase like (TTLL) family, TTLL1, 4, 5, 6, 7, 9, 11, and 13, have been demonstrated as authentic glutamate ligases that differentially catalyze the formation of the γ- (initiation) or α-linkage (elongation) (2, 3). Conversely, the shortening and removal of polyglutamate side chains are catalyzed by the 6-member cytosolic carboxypeptidase (CCP) family. Except CCP5 that specifically catalyzes removal of the branch point γ-carboxyl linked monoglutamate, the other CCPs all involve in shortening the side chain through hydrolysis of the α-carboxyl-linked glutamate (4, 5, 6, 7, 8). The homeostasis of polyglutamylation is of importance in neuronal survival (4, 9, 10, 11, 12), ciliary function (3, 13), and immune responses (14).

The substrate specificity of TTLL glutamylases was mainly determined using tubulin, the best-known substrate of polyglutamylation. The C-terminal intrinsically disordered regions of α- and β-tubulins exposed on the outer surface of microtubule (MT) are the hotspots for polyglutamylation (15, 16). With tubulins from mammalian brain or non-neuronal cell lines as the substrates, TTLL glutamylases were found not only distinguishable for their activities in initiation or elongation but also for their preference for α-tubulin or β-tubulin (2). For instance, TTLL4 and TTLL5 were characterized as initiators that prefer β-tubulin and α-tubulin respectively (2). TTLL1, 6, 11, and 13 all seemingly prefer to the elongation on α-tubulin, whereas TTLL7 catalyzes both initiation and elongation on β-tubulin (2, 17). In addition, TTLL4 is known as an exclusive initiator (18) and able to ligate glutamate to a broad range of proteins (19), such as nucleoplasmin, PELP, NAP1, and NAP2 (20, 21, 22).

Mouse brain tubulins are barely glutamylated at birth, and the polyglutamylation mainly takes place during postnatal development, with α-tubulin being glutamylated first. By the time of adult, the majority of brain tubulin is polyglutamylated (23). TTLL1 and TTLL7 are the major brain glutamylases, and knocking out either can reduce the tubulin glutamylation level in the brain (12, 24, 25, 26, 27). In contrary, the prototypic CCP—Nna1/CCP1 is the most efficient eraser that shortens the side chain and contributes substantially to the polyglutamylation homeostasis in the nervous system (4, 9, 28).

Polyglutamylation homeostasis is required for neuronal survival. Most prominently, loss of Nna1/CCP1 causes severe neurodegeneration across species (9, 29, 30, 31). In the Nna1/CCP1 mutant—*Purkinje cell degeneration* (*pcd*) mice, the tubulin polyglutamylation level in both cerebellum and cerebral cortex is elevated (4), accompanied by rapid loss of cerebellar Purkinje cells at the time of wean and progressive degeneration of selective neurons in olfactory bulb, retina, and thalamus over about a year (9, 32). Nna1 mutations also cause tetraplegia in newborn sheep (29) and damaging early-onset neurodegeneration in humans (29, 30, 31, 33). Accumulating evidence demonstrated that restoring the polyglutamylation homeostasis in *pcd* mice can prevent neuronal death. Knocking out TTLL1 could rescue the Purkinje cell loss in *pcd* mice (10, 11, 12), accompanied by a dramatic reduction in cerebellar tubulin glutamylation. Recently, we further identified that TTLL4, a β-tubulin favorable initiator, but not TTLL5, 7, or 11, also contributes to the neuronal loss in *pcd* mice. Deletion of TTLL4 did not significantly alter the tubulin glutamylation level in cerebellum but rescued the Purkinje cell death in *pcd* mice (12). Notably, although similar to TTLL4, TTLL7 also favors to modify β-tubulin, its loss did not rescue the neuronal degeneration in *pcd* mice, despite the correction of tubulin glutamylation level in cerebellum (12). This raises the question whether TTLL4 and TTLL7 behave similarly even for their common substrate—tubulin.

Previous studies showed that recombinant mammalian TTLL7 can equally modify α- and β-tubulins of free form but exhibits higher activity for MT with a preference for β-tubulin (17). The mechanism underlying this difference was revealed by analyzing a cryo-EM structure of TTLL7 in complex with MT (34). However, despite its substrate versatility and contribution to neurodegeneration in *pcd* mice, the properties of TTLL4 have been less studied.

In this study, we purified a recombinant mouse TTLL4 and characterized its enzyme properties in a pure *in vitro* system. Using an antibody-based method, we found that recombinant TTLL4 and TTLL7 exhibit distinct activity and isoform selectivity for tubulins from postnatal mouse brain. Furthermore, with synthetic peptides, which mimics the tails of α1- and β2-tubulins, the most abundant brain isoforms (35), as the substrates, we show that TTLL4 and TTLL7 exhibit different site selectivity. Their different modification sites were further confirmed with purified recombinant mouse α1A/β2A tubulin dimers (36). Interestingly, we identified a novel region in the recombinant α1A tubulin that is subjected to glutamylation by two enzymes, still at distinct sites. Finally, we showed that kinesin exhibits very different motility and affinity on MTs modified by TTLL4 and TTLL7. This study underpins the different reactivity and site specificity of TTLL4 and TTLL7 for brain tubulins, and sheds light on their distinct functions *in vivo*.

## Results

### Recombinant TTLL4 can efficiently glutamylate the newborn mouse brain tubulin

**Figure 1:**
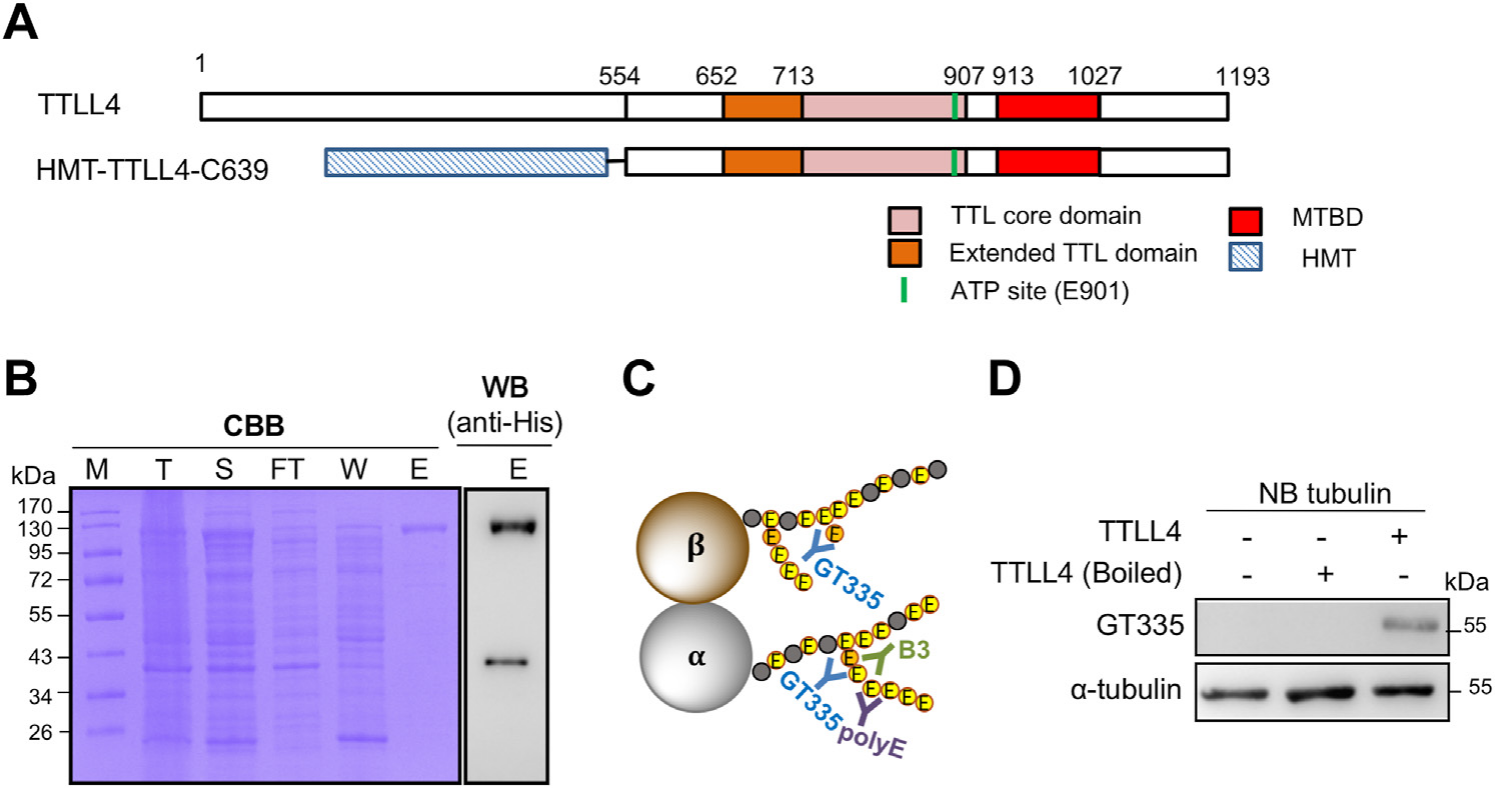
**Expression and purification of recombinant TTLL4.***A*, schematic representation of the construct of truncated TTLL4 (TTLL4-C639) (2) used in this study. The core TTL domain, extended TTL domain, microtubule-binding domain (MTBD) (34), and ATP site that are essential for TTLL4 activity are indicated in *light pink*, *orange*, *red*, and *green*, respectively. To facilitate protein purification, the truncated TTLL4 was fused with an HMT tag at the N terminus. *B*, Coomassie brilliant blue (CBB)–stained SDS-PAGE (*left panel*) showed the proportion and purity of HMT-TTLL4-C639 in the total cell lysate (T), soluble fraction (S), flow-through (FT), washing (W), and eluate (E). The major band in the eluate is immunoreactive with an anti-His antibody (*right panel*). *C*, schematic demonstration of the selectivity of GT335, B3, and polyE antibodies. *D*, glutamylation assay using tubulin purified from newborn mouse brain (NB) showed that the intact but not the heat-inactivated purified recombinant TTLL4 can increase the immunoreactivity of GT335, which recognizes the branch point glutamate of certain sites, indicative of the active enzyme. HMT, histidine-maltose binding protein-tobacco etch virus; TTLL4, tubulin tyrosine ligase like 4.

In order to characterize the enzyme properties of TTLL4, a truncated version of mouse TTLL4, which does not alter the tubulin isoform preference of the enzyme (2), was fused with a histidine-maltose binding protein-tobacco etch virus tag at its N terminus (Fig. 1*A*) and expressed using a bacterial system. Upon Coomassie brilliant blue (CBB) staining, the major band of purified protein was visualized at the predicated molecular weight (117.9 kDa) after SDS-PAGE, which was also immunoreactive with a His-tag antibody (Fig. 1*B*).

Several antibodies are available to study polyglutamylated proteins. Amongst them, GT335 is able to detect the branch point, including both monomodifications and polymodifications (37), whereas other antibodies such as B3 and polyE only recognize tubulin side chains with more than two or three glutamate residues, respectively (Fig. 1*C*) (2, 24, 38). To determine the activity of TTLL4, the recombinant enzyme was first incubated with porcine brain tubulin, and the glutamylation level was monitored using GT335 or polyE antibody, with denatured enzyme incubated at the identical condition as control. The porcine tubulin alone was immunoreactive for both GT335 and polyE antibodies, and the signals were not further increased with addition of the recombinant TTLL4 (Fig. S1*B*). We speculated that the high basal level of glutamylation already existed in this tubulin preparation might have overwhelmed the change in glutamylation level upon recombinant TTLL4 addition. Therefore, tubulins purified from newborn mice brain (NB), which are known little glutamylated, were used to test the activity of the recombinant TTLL4. Indeed, glutamylation levels of tubulin from this resource were much lower compared with those from porcine brain as evidenced by much weaker GT335 immunoreactivity (Figs. 1*D* and S1*B*). In reactions containing the intact enzyme, the GT335 signal was obviously increased compared with those with denatured enzyme or without enzyme (Fig. 1*D*). In contrast, the polyE signal remained unchanged, suggesting its inability to produce longer chain modification (Fig. S1*C*). Therefore, the recombinant TTLL4 is enzymatically active, and its activity to initiate the glutamylation of NB tubulin is readily detectable using the GT335 antibody.

### Characterization of the recombinant TTLL4

**Figure 2:**
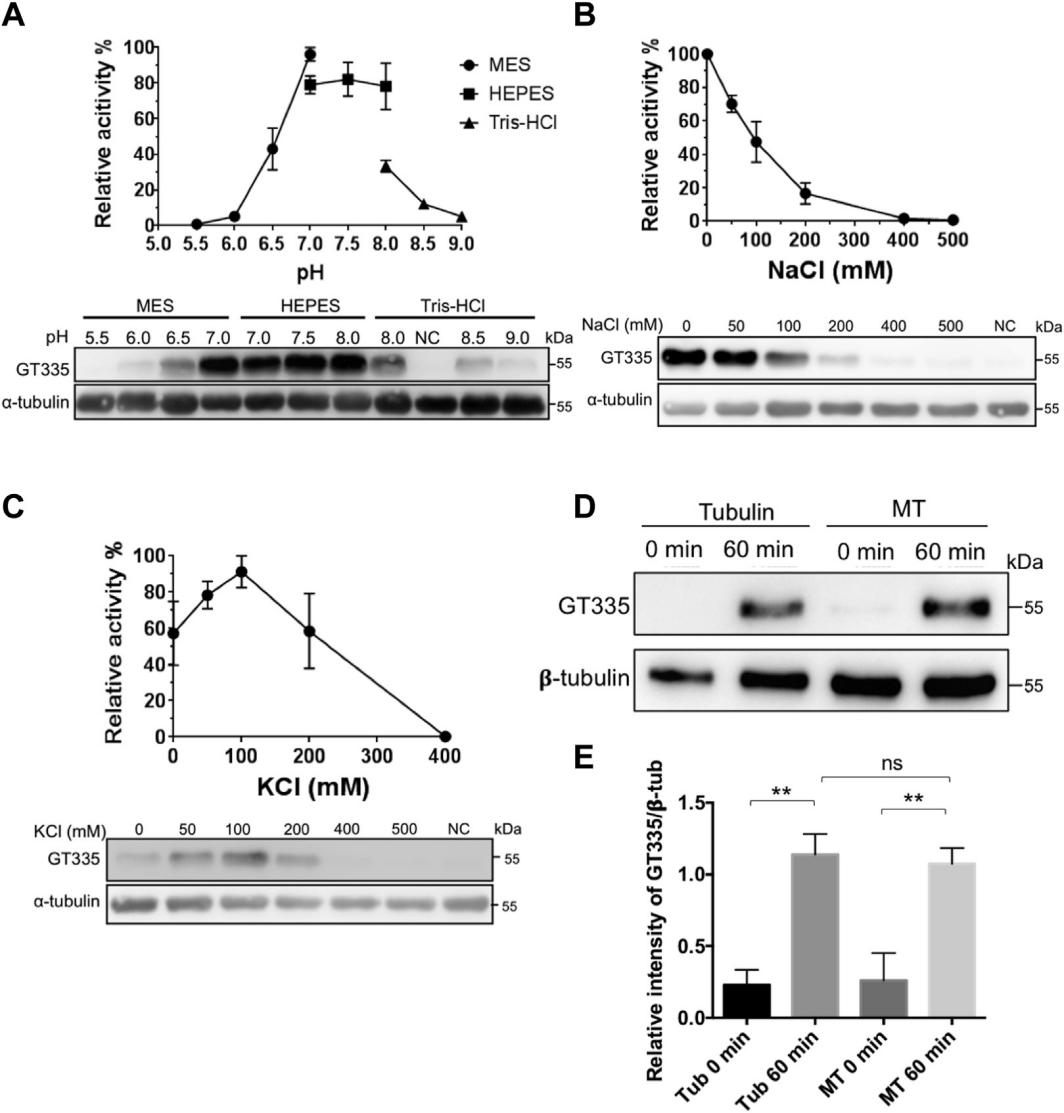
**Characterization of the property of recombinant TTLL4 with tubulin from newborn mouse brain (NB) as the substrate.** Tubulin purified from NB was incubated with purified intact or denatured (NC) recombinant TTLL4 at 37 °C for 2 h in buffers of different pH (*A*) or in the presence of different concentrations of sodium (*B*) or potassium (*C*). TTLL4 activity was measured as an increase in the intensity of GT335 signal normalized to that of corresponding α-tubulin bands with a subtraction of that in reaction with denatured enzyme (NC). *A*, the activity of the recombinant TTLL4 shows a pH-dependent manner in the range of pH 5.5 to 9.0 and is relatively stable between pH 7.0 and 8.0 in Hepes buffer. A representative image of Western blot (WB) for these conditions was shown underneath the plot. *B* and *C*, sodium (*B*) and potassium (*C*) concentration dependence of the glutamate ligase activity of recombinant TTLL4, with representative WB images shown underneath respective plots. *D*, representative blot images showing the glutamylation of free tubulins or microtubules (MTs) incubated with the recombinant TTLL4. *E*, *gray* scale quantification of results from three experiments exemplified in (*D*) showed that TTLL4 modified free tubulin and MT with comparable activity. Error bars are the mean ± SEM of triplicate determinations, ∗∗*p* < 0.01, Student's *t* test. TTLL4, tubulin tyrosine ligase like 4.

With tubulins purified from NB as the substrate, we sought to characterize the enzyme properties of the recombinant TTLL4 using an immunoblotting-based method. The pH dependency of this enzyme was analyzed in a range between pH 5.5 and 9.0. The enzyme activity increased with the pH value between 6 and 7 and remained in a comparable level in Hepes buffer between pH 7 and 8. TTLL4 activity was reduced when pH was higher than 8.5 (Fig. 2*A*).

Next, we examined how ion strength may affect the activity of the recombinant TTLL4. TTLL4 activity was reduced with increased concentration of NaCl. With the presence of 50 or 100 mM NaCl, the enzyme activity was inhibited by about 25% and 50%, respectively (Fig. 2*B*). TTLL4 activity was increased when KCl concentration was lower than 100 mM but was inhibited by KCl at higher concentrations (Fig. 2*C*). These properties are similar to those of recombinant TTLL7 (17).

The activity of TTLLs may depend on the polymerization status of tubulin (2, 17). For instance, the recombinant TTLL7 can glutamylate MTs more efficiently than free tubulins (17, 34). We wondered whether tubulin polymerization could also affect the activity of TTLL4. NB tubulins or their taxol-stabilized MTs were incubated with TTLL4 separately. We found that the free tubulin and MTs were glutamylated at similar levels after 1 h incubation (Fig. 2, *D* and *E*). Therefore, the recombinant TTLL4 can equally process free tubulins of NB and their polymerized MTs.

### Recombinant TTLL4 increased the level of GT335-immunoreactive α-isoform of NB tubulin

**Figure 3:**
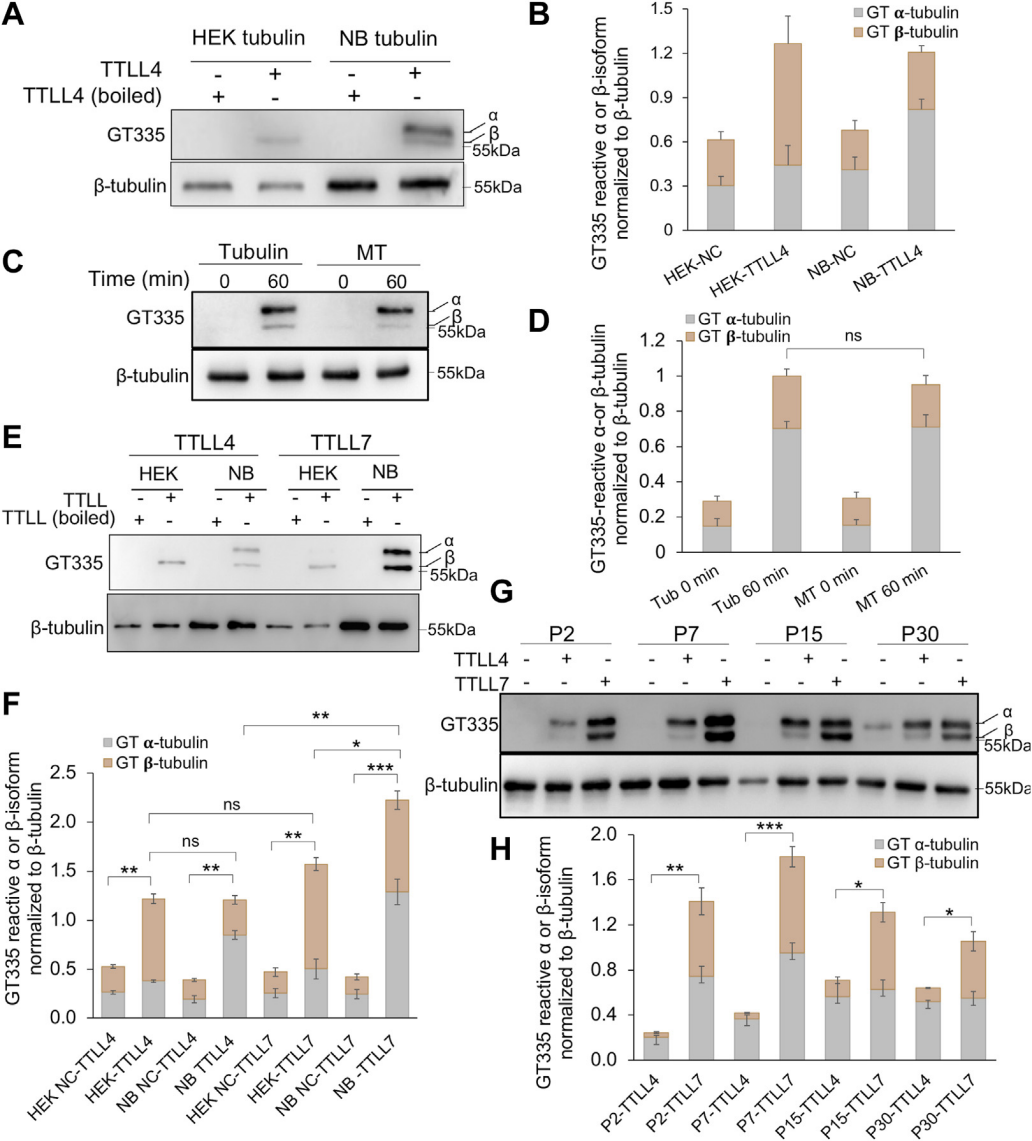
**Recombinant TTLL4 predominantly produces GT335-reactive glutamylated α-isoform for tubulins from newborn mouse brain (NB).***A*, TTLL4 glutamylation activity assay using tubulins purified from human embryonic kidney 293 (HEK293) cells or NB. Recombinant TTLL4 increased the GT335 signal of β-isoform of tubulin from HEK293 cells but predominantly produced GT335-reactive α-isoform in tubulin from NB mouse brain. *B*, quantification of four experiments shown in (*A*). TTLL4 gave rise to different GT335-reactive tubulin isoforms for HEK293 and NB tubulins (*p* < 0.05, Student's *t* test). *C*, comparison of GT335-reactive glutamylation of α- and β-isoforms of NB tubulin in a free (tubulin) or polymerized (microtubule [MT]) status after incubated with TTLL4. GT335 reactivity is higher in α-isoform than β-isoform in both free and polymerized forms of NB mouse brain tubulin. *D*, quantification of three experiments exemplified in (*C*) showed the significantly higher GT335-immunoreactive signals for α-tubulin than that for β-tubulin in both free and polymerized forms of NB tubulin (*p* < 0.01), whereas the ratios of GT335-positive β-tubulin to that of α-tubulin were not significantly different between free tubulin and MT (*p* = 0.28, Student's *t* test), suggesting polymerization did not alter the isoform preference of TTLL4. *E*, recombinant TTLL4 and TTLL7 predominantly increased the GT335 signals in β-isoform of HEK293 tubulins with similar reactivity. For NB tubulins, TTLL4 gave rise to stronger GT335 signal in the α-isoform than the β-isoforms, but TTLL7 produced comparable GT335 signal for two isoforms. TTLL7 also exhibits higher activity than TTLL4 for NB tubulin. *F*, quantification of isoform reactivity of TTLL4 and TTLL7 exemplified in (*E*). *G* and *H*, comparison of glutamylation activity between recombinant TTLL4 and TTLL7 for tubulins from developing mouse brain. For tubulin from all ages, TTLL4 gave rise to stronger GT335 signals for the α-isoform than the β-isoform, but TTLL7 produced comparable GT335 signals for two isoforms. TTLL7 exhibited higher overall activity than TTLL4 for all tubulins from all ages; error bars are the mean ± SEM of triplicate determinations. ∗*p* < 0.05, ∗∗*p* < 0.01, ∗∗∗*p* < 0.001, Student's *t* test. TTLL4, tubulin tyrosine ligase like 4; TTLL7, tubulin tyrosine ligase like 7.

GT335 antibody was generated using a peptide corresponding to the C terminus of α-tubulin with two glutamate residues in the side chain (37). It also recognizes modified β-tubulin and nontubulin substrates (2, 11, 14, 21, 37) but requires an acidic amino acid following the modified site (illustrated in Fig. 1*C* based on Refs. (26, 37)), therefore offering a tool to detect a specific subset of modified tubulins. With this antibody, we sought to determine whether the recombinant TTLL4 exhibits any tubulin isoform selectivity. To this end, a protocol to separate α- and β-isoforms of tubulin by SDS-PAGE was adopted prior to immunoblotting (39). Tubulin purified from human embryonic kidney 293 (HEK293) cells was barely immunoreactive to GT335 antibody (4, 11). After 2 h incubation with TTLL4, the glutamylation of its β-isoform was detectable (Fig. 3*A*), consistent with previous study (2). However, when TTLL4 was incubated with NB tubulin, GT335-reactive signals were detected at both α- and β-tubulin positions, with the upper band, presumably α-tubulin, being much stronger (Fig. 3, *A* and *B*).

The isoform selectivity of TTLL4 for tubulins of HEK293 cells resembles that of HeLa cells transfected with TTLL4 (2), where the GT335-immunoreactive signal for β-tubulin was stronger at the beginning of transfection and eventually reached a comparable level between isoforms after longer incubation. Unexpectedly, the GT335-immunoreactive isoform selectivity of TTLL4 for NB tubulins appeared discrepant from its previously claimed “β-tubulin preference” (2). By carefully delineating the α- and β-tubulins visualized with Ponceau S staining prior to immunostaining, we confirmed that the two GT335-reactive bands fell in the delineated positions of α- and β-tubulins, respectively, rather than two different populations in the same isoform (data not shown).

To determine whether the isoform selectivity of GT335-immunoreactive products varies during reaction, we monitored the glutamylation time course of tubulin incubated with the recombinant TTLL4. The GT335 immunoreactive α-tubulin-like signal was detectable in the first 5 min after reaction and rapidly increased in the first 30 min. After 1 h incubation, this signal reached the highest level and was no longer increased with further incubation (Fig. S2*A*). In contrast, the GT335-immunoreactive β-tubulin-like signal was hardly detectable in the first 15 min and became obvious after 30 min incubation (Fig. S2*A*). Further incubation only slightly increased the GT335-positive β-tubulin-like signal but still much weaker than that of α-tubulin band (Fig. S2*A*). Although GT335 antibody selectively recognizes a subset of glutamylated tubulin isoforms and is more sensitive to α-tubulin (26, 37), our results suggested that at least a subset of α-tubulin was modified by the recombinant TTLL4.

### Recombinant TTLL4 similarly increased the level of GT335-immunoreactive α-isoform in both free NB tubulins and MTs

TTLL polyglutamylases may exhibit different isoform preference for free tubulins and MTs. For instance, although recombinant TTLL7 can equally process α- and β-isoforms of free tubulins, it favors the β-isoform in the polymerized form—MTs (17, 34). We wondered whether the polymerization status of tubulins could also affect the tubulin isoform selectivity of recombinant TTLL4. The free NB tubulins and their polymerized MTs were incubated with the recombinant TTLL4 respectively, and the glutamylation levels of α- and β-isoforms were monitored. Similar to the free tubulins, in the MTs incubated with TTLL4, the GT335-immunoreactive signal at the α-form position is also significantly stronger than that of the β-form (Fig. 3, *C* and *D*).

To exclude the possibility that the affinity chromatography–mediated tubulin preparation might lead to the loss of certain TTLL4-favored β-isoforms, we tested the GT335-immunoreactive pattern of MTs prepared using a traditional taxol-driven polymerization method (40) that were treated with TTLL4. Similarly, the GT335 immunoreactivity is still stronger at the α-isoform band (Fig. S2*B*). Taken together, the recombinant TTLL4 modifies both α- and β-isoforms of the NB tubulin and gives rise to stronger GT335 immunoreactivity for α-isoform product than that for the β-isoform in both free tubulins and MTs.

### The recombinant TTLL4 and TTLL7 differ in isoform selectivity for tubulins from NB

We were curious whether TTLL7, another β-tubulin-favorable glutamylase, also gives rise to similar GT335-reactive isoform selectivity for tubulins. When a recombinant TTLL7 purified from bacteria (Fig. S3*A*) was incubated with HEK tubulins, the GT335-immunoreactive signals were stronger at the β-tubulin position than that at the α-tubulin position, similar to TTLL4 (Fig. 3, *E* and *F*). However, when TTLL7 was incubated with NB tubulins, the GT335 signals at α- and β-tubulin positions were increased to a similar level (Fig. 3, *E* and *F*), and the intensities in two isoforms kept being comparable since 3 min after reaction (Fig. S3*B*). Notably, in tubulins from HEK cells, the levels of GT335-positive products generated by TTLL4 and TTLL7 were not significantly different, but TTLL7 gave rise to much stronger GT335 signals for tubulins from NB than TTLL4 (Fig. 3, *E* and *F*). Therefore, TTLL4 and TTLL7 predominantly modify the β-isoform of tubulin from HEK cells. For tubulins from NB, TTLL7 exhibits similar reactivity for both isoforms, consistent with previous observation (17), whereas TTLL4 gives rise to stronger glutamylation immunosignals in α-tubulin than that in the β-isoform.

### The recombinant TTLL4 and TTLL7 exhibit different isoform selectivity in glutamylation of tubulins of postnatal developing mouse brain

Different tubulin isoforms are expressed in distinctive temporal patterns during mouse brain development (41). Using GT335 immunoreactivity as a readout, we wonder whether TTLL4 could exhibit different tubulin isoform selectivity in the developing mouse brain. Tubulins from mouse brain of different postnatal developing stages were purified, for which GT335 antibody mainly recognizes the α-isoform, and the glutamylation levels increase with age, consistent with previous reports (4). Incubation with the recombinant TTLL4 led to an increase in glutamylation level of tubulin from all ages examined, indicative of the existence of TTLL4 substrates in all these tubulin preparations (Fig. 3*G*). TTLL4 exhibits higher overall activity to brain tubulins from elder (P15 and P30) mice than those from younger (P2 and P7) mice, but it was consistently more selective to the α-isoform than the β-isoform in tubulins from all ages examined (Fig. 3, *G* and *H*).

Incubation with TTLL7 also increased the glutamylation levels in brain tubulins from different ages, with GT335 immunoreactivities for α- and β-isoforms being comparable in all these preparations (Fig. 3, *G* and *H*). This is consistent with previous reports that TTLL7 similarly glutamylates α- and β-isoforms of free tubulins (17). Different from treatment with the recombinant TTLL4, tubulins purified from younger mouse brains were more reactive to TTLL7 than those from P30 brain. In comparison, TTLL7 exhibited much higher overall activities to brain tubulins from all ages than TTLL4 (Fig. 3, *G* and *H*). Taken together, TTLL7 appears generally more active to the brain tubulins than TTLL4. TTLL7 does not exhibit isoform preference for free brain tubulins from different ages, but TTLL4 is more selective for the α-isoform to generate GT335-positive signals.

One possibility for the distinct age-dependent reactivities of postnatal brain tubulin to TTLL4 and TTLL7 could be that the modifications by individual endogenous TTLLs already exist to different extents during development, making them differentially sensitive for further modification by respective recombinant TTLLs. We examined the temporal expression of three glutamylation initiators, that is, *Ttll4*, *Ttll5*, and *Ttll7*, in developing brain using quantitative RT–PCR. *Ttll4* is expressed in the highest level at the birth of mice and remains in a reduced level after P7 (Fig. S4*A*), whereas the expression of *Ttll7* is fairly consistent in the postnatal developing brain (Fig. S4*C*). Therefore, the endogenous expression level of *Ttll4* or *Ttll7* unlikely contributed to the distinct age-dependent competence of tubulins to respective recombinant enzymes.

Interestingly, the expression of *Ttll5*, known as an α-initiator, is relatively low before P7 but increased about a fold after P15 (Fig. S4*B*). As TTLL4 can modify at least a subset of α-tubulin, the complementary temporal expression of TTLL4 and TTLL5 might reflect the predominant α-tubulin initiators at different developmental stages.

### Recombinant TTLL7 but not TTLL4 exhibits elongation activity on mouse brain tubulins

**Figure 4:**
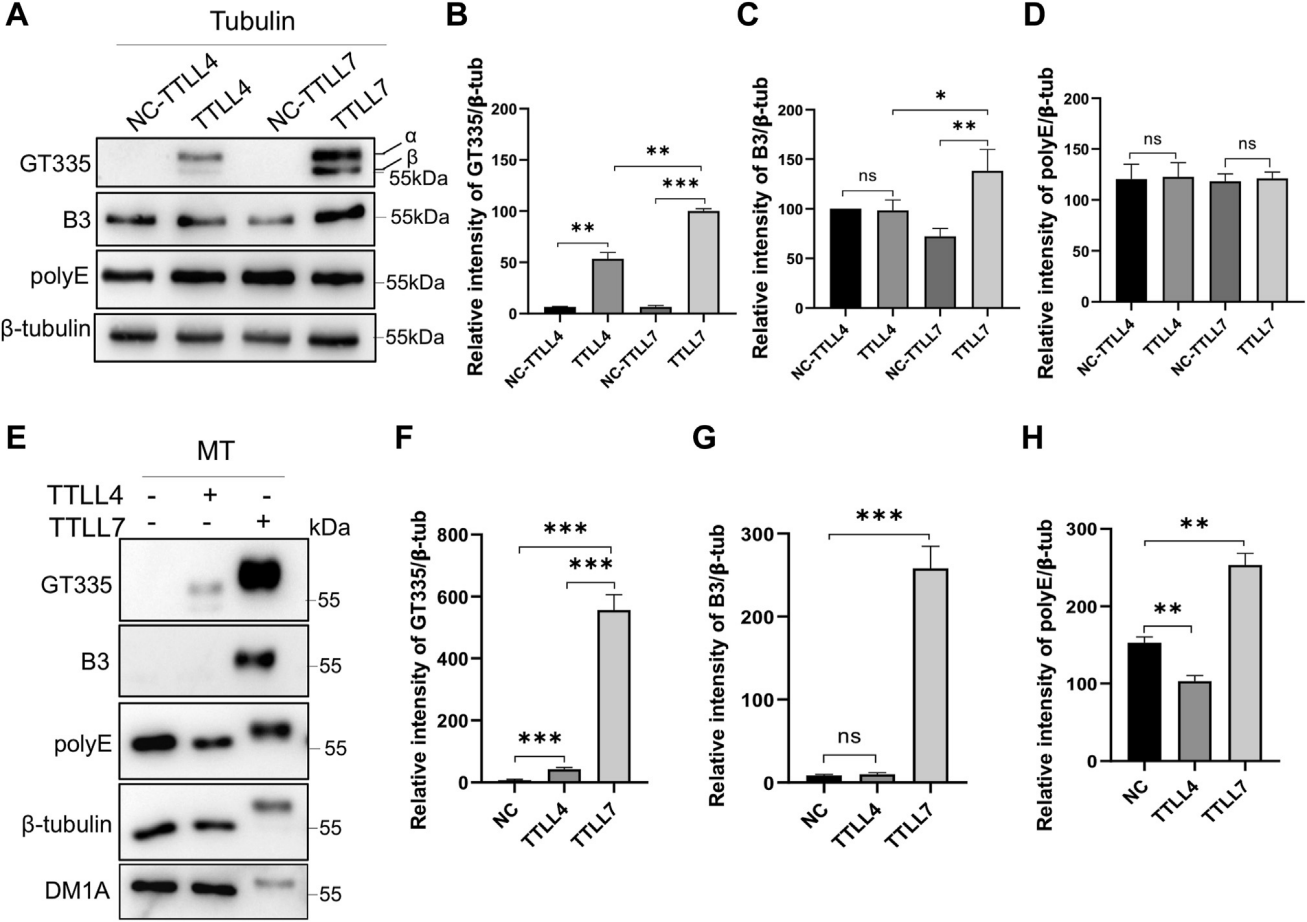
**Recombinant TTLL7 but not TTLL4 possesses the elongation activity for brain tubulins.***A*, representative images showing that newborn mouse brain (NB) tubulins incubated with recombinant TTLL4 or TTLL7 with their initiation activities monitored with GT335 antibody and the elongation activities determined with B3 (which recognizes >2 glutamate in the side chain) or polyE (which recognizes >3 consecutive glutamate residues). Compared with the control, TTLL4 only increased the signal of GT335, but neither that of B3 nor polyE, suggesting its sole initiation activity. Whereas, TTLL7 increased signals of both GT335 and B3, but not that of polyE, suggesting that it also produces short chains on tubulins. The quantifications of triplicate experiments are shown in (*B*–*D*). *E*, microtubules (MTs) polymerized from NB tubulins were incubated with TTLL4 or TTLL7 to examine their initiation activity with GT335 or elongation activity with B3 and polyE antibodies. While TTLL4 only increased the GT335 signal, TTLL7 increased the signals of all three antibodies, suggesting that TTLL4 can only initiate the side chain, but TTLL7 is able to generate the long side chains. Notably, the immunosignals of both β-tubulin and α-tubulin (DM1A) exhibit a slow-migration shift in TTLL7 modified MTs, suggesting a possible modification on both subunits. Quantification from triplicate experiments was shown in (*F*–*H*), respectively. Error bars are the mean ± SEM of triplicate determinations. ∗*p* < 0.05; ∗∗*p* < 0.1; ∗∗∗*p* < 0.001, Student' *t* test. TTLL4, tubulin tyrosine ligase like 4; TTLL7, tubulin tyrosine ligase like 7.

We further examined whether TTLL4 and TTLL7 can elongate the glutamate side chain of tubulins using B3 and polyE antibodies, which recognize more than two or three glutamate residues in the side chain of α-tubulin respectively (Fig. 1*C*). For NB tubulins, TTLL7, but not TTLL4, increased the B3 signals. However, neither enzymes altered the polyE signals (Fig. 4, *A*–*D*), consistent with previous report that TTLL4 is an initiator (2, 18).

The recombinant TTLL7 can efficiently extend the glutamate side chains in MTs (17, 34). Indeed, with the stabilized MTs as the substrate, TTLL7 significantly increased the signals of both B3 and polyE (Fig. 4, *E*–*H*). Notably, the immunosignals of both β-tubulin and α-tubulin (DM1A) in these reactions showed a slow-migration shift, suggesting a probable addition of long glutamate side chains on both subunits. In contrast, TTLL4 only increased the signal of GT335, further reinforced its role of an initiator.

### Recombinant TTLL4 and TTLL7 modify different sites in peptides corresponding tubulin tails

Given the site selectivity of GT335 antibody, the different competence of α- and β-tubulins to generate GT335-positive population upon TTLL4 and TTLL7 treatment may result from the different immunoreactivities of respective sites modified by two enzymes. To this end, we synthesized the peptides corresponding to respective C-terminal tails of the detyrosinated α1A/B- and β2A/B- (a.k.a. βII) tubulins, the most abundant isoforms in the brain (35, 42), and incubated them individually with the recombinant TTLL4 or TTLL7 in the presence of [^13^C5]-glutamate, which allows to distinguish the incorporated glutamate from those in the peptides.

**Figure 5:**
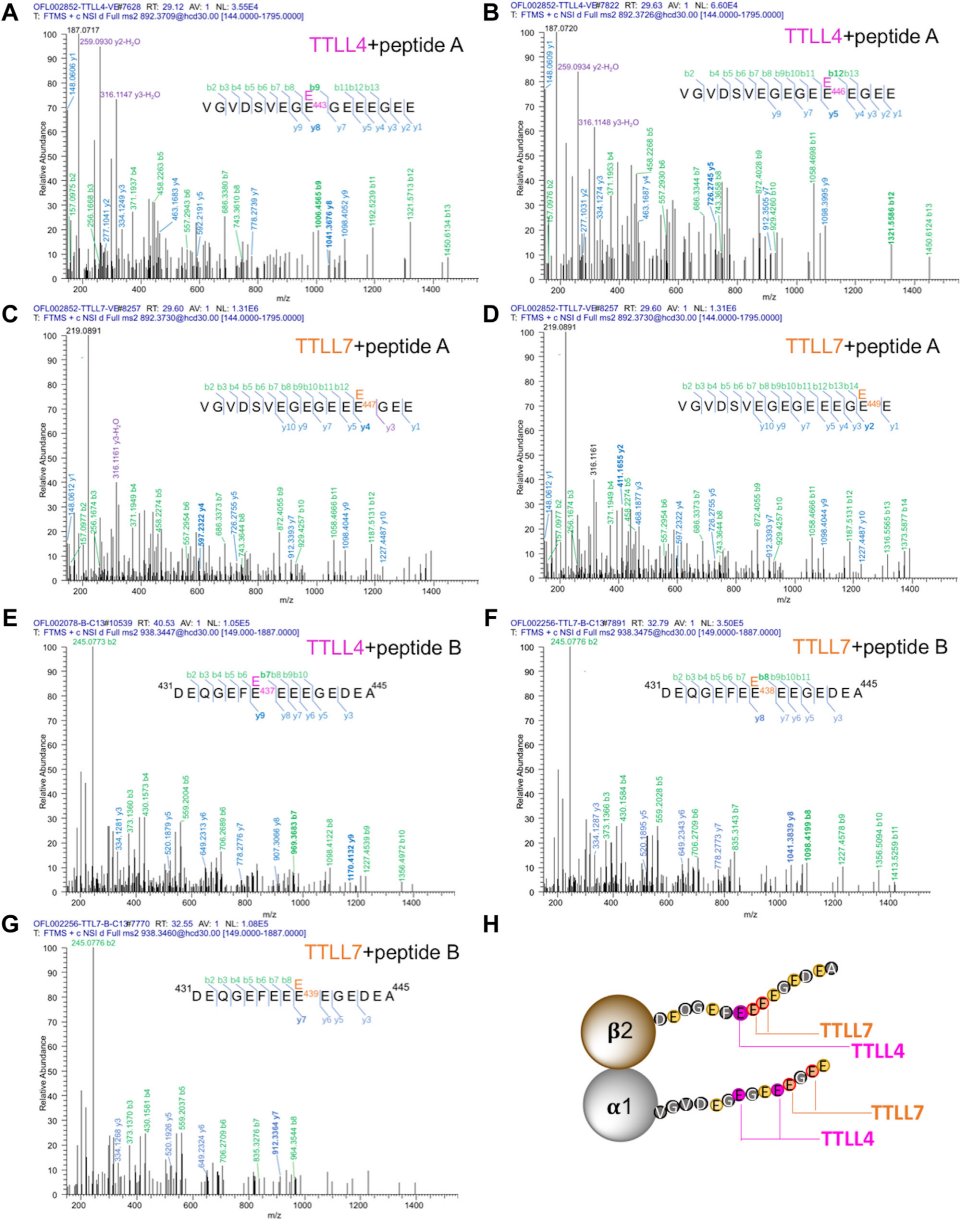
**MS/MS sequencing of the peptide corresponding to the α1-and β2-tubulin tails glutamylated by recombinant TTLL4 or TTLL7.***A*–*D*, MS spectra assignment for synthetic peptide corresponding to the α1-tubulin tail (peptide A) incorporated one [^13^C5]-glutamate after incubation with the recombinant TTLL4 (*A* and *B*) or TTLL7 (*C* and *D*). The isotope-labeled glutamate residues were identified on E443 (*A*) and E446 (*B*) respectively in the peptide modified by TTLL4, but on E447 (*C*) and E449 (*D*) in the peptide modified by TTLL7. *E*–*G*, MS spectra assignment for the synthetic peptide corresponding to the β2-tubulin tail (peptide B) incorporated one [^13^C5]-glutamate after incubation with the recombinant TTLL4 (*E*) or TTLL7 (*F* and *G*). The isotope-labeled glutamate is only identified on E437 in TTLL4-modified peptide (*E*), but on E438 (*F*) and E439 (*G*) in the peptide modified by TTLL7. Identified b- and y-ion series and the amino acid sequence corresponding to each spectrum are indicated, with those characteristic for the respective modified sites labeled in *bold*. *H*, an illustration showing the different modification sites by two enzymes on peptide A and peptide B. MS, mass spectrometry; TTLL4, tubulin tyrosine ligase like 4; TTLL7, tubulin tyrosine ligase like 7.

Assessed with LC–MS, the +1E forms of both peptides predominated in the products of all reactions with active enzymes, whereas the ions corresponding to the mass of these products were hardly detectable in the control groups (Fig. S5). For the α1-tubulin peptide, the recombinant TTLL7 could generate the +1E product with ∼8% conversion rate, whereas the +1E production in the TTLL4-catalyzed reaction was about 40 times less (Fig. S5*A*). Despite the low production of +1E α1-tail peptide by TTLL4, tandem mass spectrometry (MS/MS) analysis unambiguously confirmed the incorporation of ^13^C5-labeled glutamate at E^443^ and E^446^ with both y and b ions characteristic for these modifications being identified (Fig. 5, *A* and *B*). However, in the TTLL7-modified +1E α1-peptide, the ^13^C5-labeled glutamate was identified on E^447^ and E^449^, but no convincing incorporation could be determined on E^443^ or E^446^ (Fig. 5, *C* and *D*). These results suggested that TTLL4 and TTLL7 select different sites on the α1-tubulin tail peptide.

For the β2 tail peptide, two enzymes gave rise to similar conversion rates between 2% and 4% (Fig. S5*B*). Furthermore, MS/MS analysis revealed that in the TTLL4-modified +1E product, the ^13^C5-labeled glutamate residue was only identified on E^437^ (Fig. 5*E*). In contrast, in the +1E product from TTLL7 reaction, the ^13^C5-labeled glutamate residue could be identified on both E^438^ and E^439^ (Fig. 5, *F* and *G*). Notably, we never observed a modification on E^437^ in the products of TTLL7. These results again indicated that TTLL4 and TTLL7 also preferentially modify distinctive sites on β2 tubulin tail, whereas TTLL4 exhibits higher selectivity.

### TTLL4 and TTLL7 modify the C-terminal tail of a recombinant α1A-tubulin at different sites

**Figure 6:**
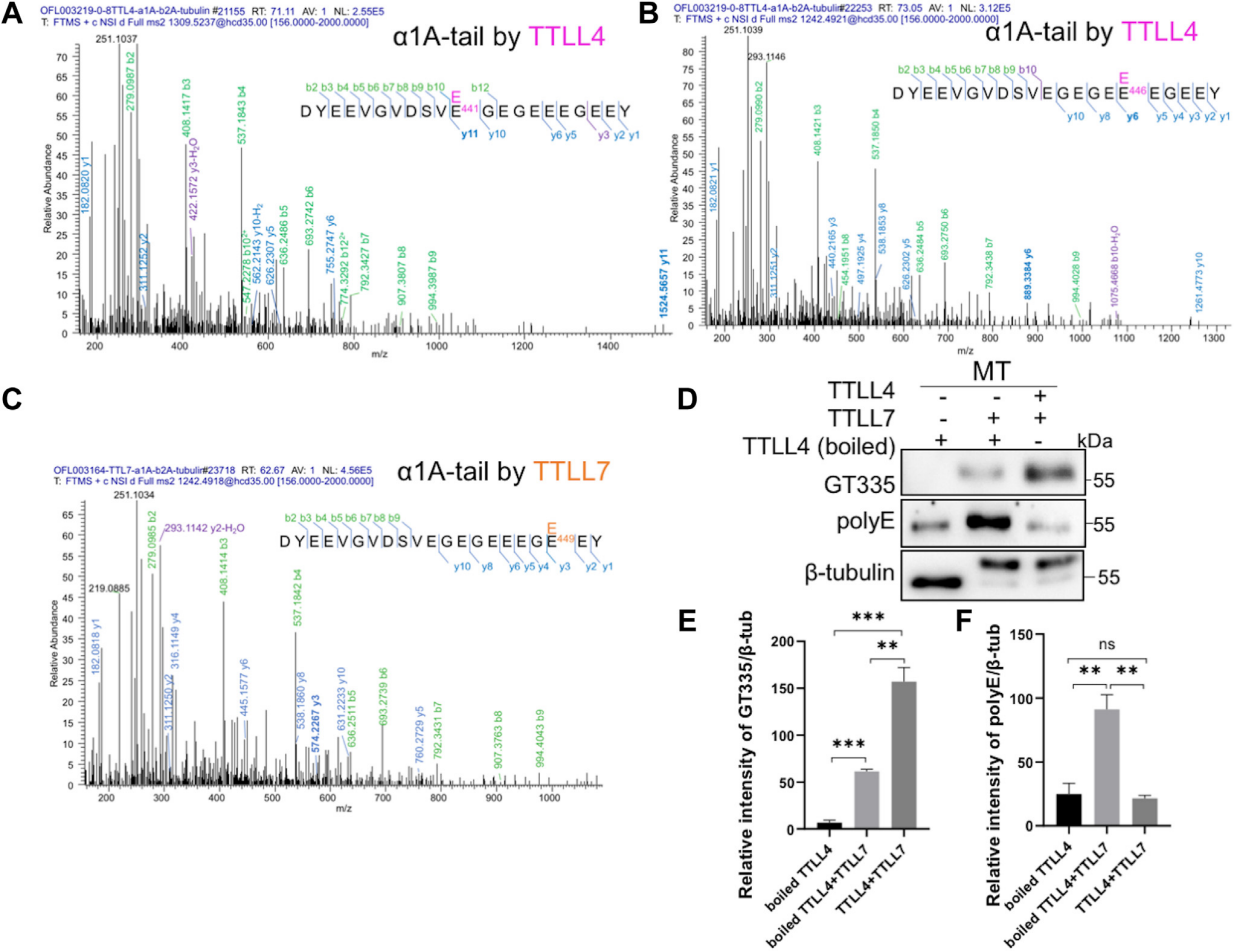
**The incompatible modification sites of TTLL4 and TTLL7 in the C-terminal tail of tubulins.***A* and *B*, MS spectra assignment for the tryptic C-terminal peptide of the recombinant α1A (36) incorporated with one [^13^C5]-glutamate after incubation with TTLL4. The isotope-labeled glutamate residues were identified on E441 (*A*) and E446 (*B*), respectively. *C*, MS spectra assignment for the tryptic C-terminal peptide of the recombinant α1A incorporated with one [^13^C5]-glutamate after incubation with TTLL7. The isotope-labeled glutamate can only be convincingly detected on E449. Identified b- and y-ion series and the amino acid sequence corresponding to each spectrum are indicated, with those characteristic for the respective modified sites labeled in *bold*. *D*, microtubules (MTs) of new born mouse brain were preincubated with denatured or intact TTLL4 with a ratio of enzyme to tubulins of 1:2 for 2 h and then supplemented with TTLL7 (with a ratio of enzyme to tubulins of 1:10 because of the high activity of TTLL7). The initiation and elongation activities are monitored using GT335 and polyE antibodies respectively. Preincubation with TTLL4 did not block TTLL7 for creating more initiation sites but reduced the elongation activity of TTLL7. *E* and *F*, quantification of normalized GT335 signals (*E*) or polyE signals (*F*) from three experiments. ∗∗*p* < 0.01; ∗∗∗*p* < 0.001, ns, no significance, Student's *t* test. TTLL4, tubulin tyrosine ligase like 4; TTLL7, tubulin tyrosine ligase like 7.

To determine whether TTLL4 and TTLL7 also modify different sites on tubulins, we first attempted to analyze their modification sites on purified brain tubulins. However, we were not successful in challenging the known difficulties to detect the modified sites on brain tubulins because of the complexity of mixed isoforms, the existence of various post-translational modifications, and unfavorable ionization of negatively charged peptides (43, 44). As an alternative, a purified functional recombinant α1A/β2A dimer (36) was used as the substrate to analyze the modification sites of TTLL4 and TTLL7. In this assay, the α1A C-terminal tail generated after trypsin digestion can be convincingly detected, and the [^13^C5]-glutamate incorporation in this peptide was identified in both reactions with TTLL4 and TTLL7. MS/MS analysis revealed two ions of the tryptic tail peptide with +1E modification in TTLL4-treated tubulins, and the modification sites were localized on E^441^ and E^446^, respectively (Fig. 6, *A* and *B*). In the +1E tail peptide produced by TTLL7, the modification was determined on E^449^ (Fig. 6*C*). These results partially recapitulated their individual modification sites identified on the corresponding synthetic peptides (Fig. 5) and confirmed the different site selections by TTLL4 and TTLL7 on the C-terminal tail of α1 tubulin. Unfortunately, the C-terminal tails of β2 tubulin were not identified even in the unmodified form, probably because of the large fragment generated after trypsin digestion. Given that the recombinant β2 tubulin contains a FLAG tag at its C terminus (36), which may affect the modifications by TTLLs anyway, we did not further pursue the modifications on β2 tails with this preparation.

The different site selectivity of TTLL4 and TTLL7 made us wonder whether tubulin glutamylation initiated by one enzyme can affect the initiation or elongation activity of another. As TTLL7 can both initiate and elongate the side chain on MTs, we tested how glutamylation of MTs by TTLL4 affects TTLL7 for further initiation or elongation. To this end, MTs were first incubated with the denatured or intact TTLL4 at 37 °C for 2 h, when the enzyme had become inactive (data not shown), and then TTLL7 was supplemented to the reaction. Assessed with GT335 antibody, the total branch-point glutamylation level was higher in MTs that were first incubated with active TTLL4 than that preincubated with the denatured enzyme, suggesting the additive initiation by TTLL7 (Fig. 6, *D* and *E*). Compared with the control, TTLL7 significantly increased the polyE signals in MTs preincubated with denatured TTLL4. However, the ability of TTLL7 to increase the polyE signals was significantly attenuated by preincubation with the intact TTLL4 (Fig. 6, *D* and *F*), suggesting that the initiation of the side chains by TTLL4 may hurdle TTLL7 for further elongation.

### Novel glutamylation sites identified in the recombinant α1A-tubulin

**Figure 7:**
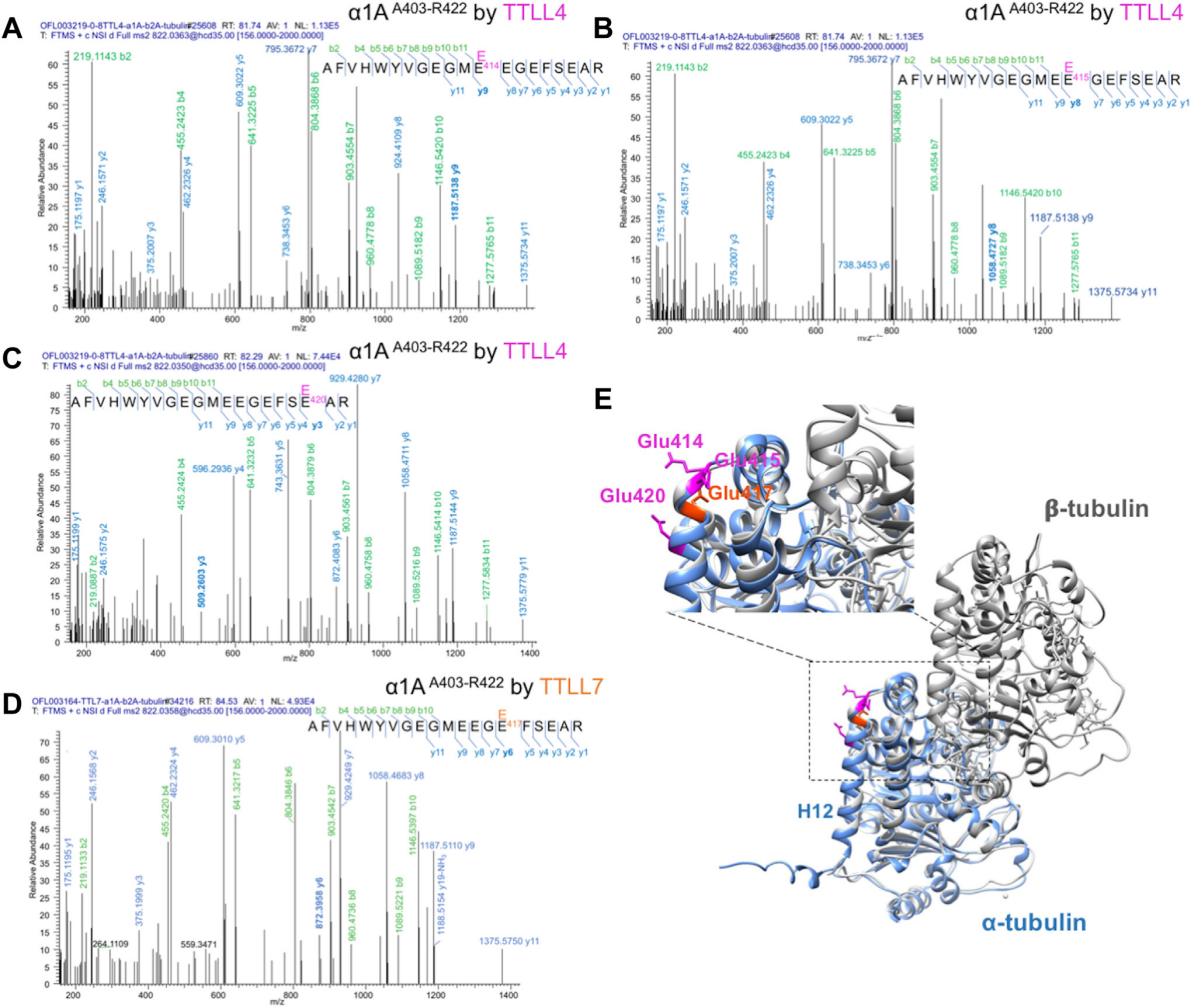
**MS/MS sequencing of a novel α1A-tubulin region glutamylated by recombinant TTLL4 or TTLL7 in a recombinant α1A/β2 dimer.***A*–*C*, MS spectra assignment for the tryptic peptide A^403^–R^422^ in the recombinant α1A-tubulin incorporated with one [^13^C5]-glutamate after incubation with TTLL4. The isotope-labeled glutamate residues were identified on E414 (*A*), E415 (*B*), and E420 (*C*), respectively. *D*, MS spectra assignment for the tryptic peptide A^403^–R^422^ in the recombinant α1A-tubulin incorporated with one [^13^C5]-glutamate after incubation with TTLL7. The isotope-labeled glutamate can only be convincingly detected on E417. Identified b- and y-ion series and the amino acid sequence corresponding to each spectrum are indicated, with those characteristic for the respective modified sites labeled in *bold*. *E*, demonstration of the locations of newly identified glutamylation sites in α1A-tubulin. The mouse α1A tubulin structure predicted by AlphaFold (identifier: AF-P68369-F1-model_v4 (50, 51) [*blue*] was superposed with that of bovine α1A/β2B dimers [Protein Data Bank ID: 1JFF (43), in *gray*]). The glutamate residues modified by TTLL4 and TTLL7 are highlighted in *pink* and *orange*, respectively. MS/MS, tandem mass spectrometry; TTLL4, tubulin tyrosine ligase like 4; TTLL7, tubulin tyrosine ligase like 7.

Interestingly, in the TTLL-treated recombinant α1A-tubulin dimers, an additional tryptic peptide (residues A^403^–R^422^) with the incorporation of [^13^C5]-labeled glutamate was identified. This peptide is located near the C-terminal tail of α1A-tubulin (Fig. 7, *A*–*D*). Particularly, the glutamylation was localized on E^414^, E^415^, and E^420^ in TTLL4-treated tubulins (Fig. 7, *A*–*C*), whereas only E^417^ modification was identified in TTLL7-treated tubulins (Fig. 7*D*). These residues are located in the loop between helices H11 and H12 (E^414^, E^415^) or the N terminus of H12 (E^417^, E^420^; Fig. 7*E*) of α1 tubulin, a region facing to the β-tubulin on the outer surface of MTs (Fig. 7*E*) (43). Despite the adjacent locations of these residues, they are differently selected by TTLL4 and TTLL7, further emphasizing the different site selectivity between these two enzymes.

### Tubulin modifications by TTLL4 and TTLL7 differently affect the interaction between MTs and kinesin-1

**Figure 8:**
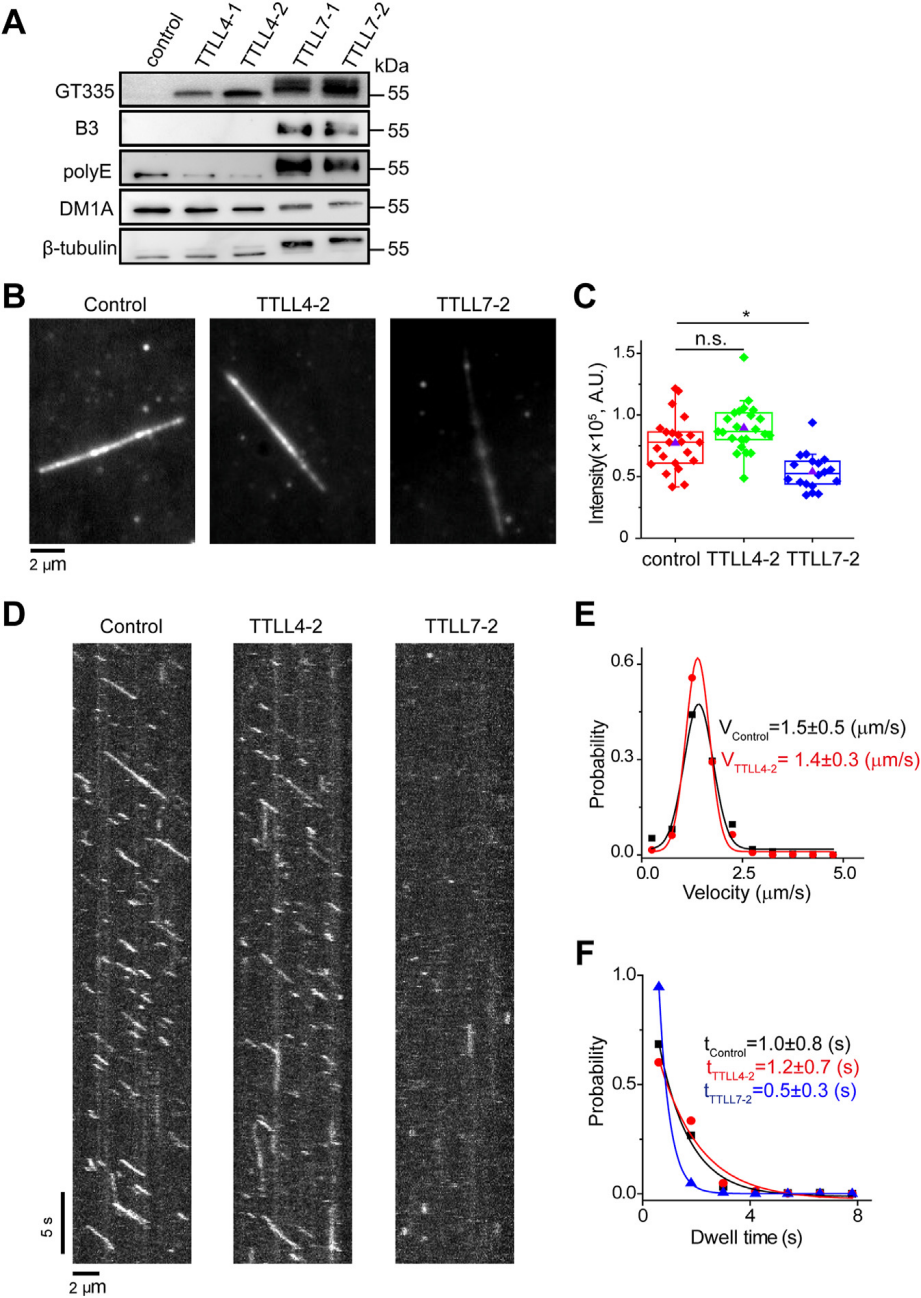
**Tubulin polyglutamylation affects the interaction between microtubules (MTs) and kinesin-1.***A*, immunoblotting images showed that MTs incubated with the low concentration of enzymes (TTLL4-1 or TTLL7-1; in a ratio of enzyme to tubulins of 1:10) or high concentration of enzymes (TTLL4-2 or TTLL7-2; in a ratio of enzyme to tubulins of 1:2) are modified by respective enzymes. Given the similar results using MTs modified with two concentrations of individual enzymes, only those using high concentration enzymes were presented below. *B*, representative projection images of Kif5c-GFP binding to control, TTLL4-treated, and TTLL7-treated MTs. To quantify the interaction between Kif5c and MTs, we recorded the binding events on MTs and measured the summation of fluorescence intensity over a period (1200 frames, 100 ms per frame with an exposure time of 50 ms). *C*, statistical comparison of fluorescence intensity summation of Kif5c-GFP on control (*red*, n = 22 MTs from three experiments), TTLL4-treated (*green*, n = 23 MTs from three experiments), and TTLL7-treated MTs (*blue*, n = 18 MTs from three experiments). The statistical analysis was performed using two-tailed Mann–Whitney *U* test with Bonferroni correction, n.s., no significance; ∗*p* < 0.05. *D*, representative kymographs of single-molecule Kif5c-GFP binding to control, TTLL4- or TTLL7-treated MTs. *E*, the distribution of the velocity of Kif5c-GFP on control (*black*, *V*_control_ = 1.5 ± 0.5 μm/s, n = 854 binding events from three experiments) and TTLL4-treated MTs (*red*, *V*_TTLL4_ = 1.4 ± 0.3 μm/s, n = 1205 binding events from three experiments). Kif5c-GFP on TTLL7-treated MTs barely moves, so we did not count its velocity on TTLL7-treated MTs. The distribution of the velocity of Kif5c-GFP on TTLL4-treated MTs was similar to that on the control. Statistical analysis was performed using two-tailed Mann–Whitney *U* test with Bonferroni correction. *F*, the distribution of dwell time of Kif5c-GFP on control (*black*, *t*_control_ = 1.0 ± 0.8 s, n = 854 binding events from three experiments), TTLL4-treated (*red*, *t*_TTLL4_ = 1.2 ± 0.3 s, n = 1205 binding events from three experiments), and TTLL7-treated MTs (*blue*, *t*_TTLL7_ = 0.5 ± 0.3 s, n = 187 binding events from three experiments). TTLL4, tubulin tyrosine ligase like 4; TTLL7, tubulin tyrosine ligase like 7.

We wondered how tubulin modifications by TTLL4 and TTLL7 could affect the interaction between MTs and regulatory proteins. Therefore, we measured the binding and motility of Kif5c (a rat isoform of kinesin-1), as an example, on MTs treated by either TTLL4 or TTLL7. Immunoblotting analysis confirmed the modifications of MTs by TTLL4 and TTLL7 (Fig. 8*A*). We found that the binding of Kif5c on TTLL7-treated MTs was significantly weaker than that on control MTs (Fig. 8, *B* and *C*). In agreement with this, much fewer numbers of Kif5c were observed on TTLL7-treated MTs in our single-molecule imaging experiments (Fig. 8*D*). Moreover, for those bound on MTs, the dwell time was significantly shorter compared with the control (Fig. 8*F*). These results demonstrate that tubulin polyglutamylation generated by TTLL7 weakens the interaction between MTs and Kif5c. Moreover, Kif5c-GFP on TTLL7-treated MTs barely moves (Fig. 8*D*). On the contrary, the modification from TTLL4 only showed moderate effects on the binding, motility, and dwell time of Kif5c on MTs (Fig. 8, *C*–*F*). The aforementioned results suggest that the distinct tubulin polyglutamylation patterns generated by TTLL4 and TTLL7 have different effects on the interaction between MTs and kinesin-1.

## Discussion

Polyglutamylation homeostasis is required for the survival of selective neurons. Mutations of its prototypic eraser Nna1/CCP1 cause devastating neurodegeneration in humans, mouse, and sheep (9, 29, 30, 31, 32). Loss of polyglutamate chain elongator TTLL1 can correct the hyperglutamylation and rescue neuronal death in *pcd* mice, mutants of Nna1/CCP1 (10, 11, 12), emphasizing the functional importance of the balance between glutamate addition and elimination. Recent studies further showed that despite their similar tubulin isoform preference, deletion of the initiator TTLL4 (12), but not TTLL7 (12, 26), can also rescue the neuronal death in *pcd* mice. In order to understand the differential properties and functions of TTLL4 and TTLL7, here we purified a recombinant mouse TTLL4 and characterized its properties with brain tubulin as the substrate using an antibody-based method. We show that for brain tubulins, TTLL4 gave raise to stronger glutamylation signals in the α-isoform than in the β-isoform, but TTLL7 produced comparable glutamylation signals between two isoforms. Consistent with previous findings, TTLL7, but not TTLL4, can efficiently elongate the glutamate side chains on MTs. Given the site selectivity of the glutamylation antibody (GT335) (26, 37), we further pursued the modification site specificity of these two enzymes. Interestingly, TTLL4 and TTLL7 exhibit distinct site selectivity in synthetic peptides mimicking the C-terminal tails of α1A- and β2 tubulins as well as a recombinant tubulin. In addition, mouse brain MTs modified by TTLL4 are less favorable for TTLL7 to further elongate, suggesting that its elongation activity is affected by the modifications with TTLL4. Finally, we showed that kinesin behaves differently on MTs modified by two enzymes. This study revealed the distinguished activity, site selectivity, and function between TTLL4 and TTLL7 and pinpointed the possible crosstalk between two glutamylases in regulating modifications of brain tubulin.

The tubulin isoform preference of TTLLs can be measured based on the incorporation of radioactive isotype–labeled glutamate in each isoform after SDS-PAGE using a special gel composition that differentiates the migration behavior of α- and β-tubulins (2, 17). However, this method does not allow to observe the glutamylated isoforms of a specific subset. The selectivity of GT335 antibody rendered a tool to evaluate the glutamylated tubulin of a specific set in either isoform. Previously, TTLL4 has been identified as a glutamylase that prefers β-tubulin (2). With HEK293 cell tubulin as the substrate, we recapitulated this conclusion through detection of the GT335 immunoreactivity. However, in brain tubulins, we found that TTLL4 gives rise to much stronger GT335 signal for the α-isoform than the β-isoform. Although GT335 immunoreactivity does not necessarily represent all the modified substrates, these results still manifest that a non-negligible subset of α-tubulin in the mouse brain is subjected to modification by TTLL4. This observation was further confirmed by the MS analysis with peptide mimicking α-tubulin tail and a recombinant mouse α-tubulin. Its high site selectivity on β2-tail (Fig. 5*E*) may account for the weak GT335 immunoreactivity of the β-isoform product generated by TTLL4.

This study revealed differences between the reactivities of TTLL4 and TTLL7 in several aspects. First, using the antibody-based method, we recapitulated that TTLL7 is both a glutamylation initiator and an elongator. It can generate short chain on tubulins and long chain on MTs. In contrast, TTLL4 is an absolute initiator for both free tubulins and MTs, consistent with previous report (18). This property of TTLL4 seemingly complies with its activity for many other nontubulin substrates that do not form polymers. Second, for brain tubulins, TTLL4 gives rise higher glutamylation immunosignals in the α-isoform than the β-isoform, but TTLL7 exhibits equal reactivity to the two isoforms. Third, the recombinant TTLL7 generally exhibits much stronger activity than TTLL4, especially for tubulins purified from younger mice, consistent with its role as a major glutamylase in the brain (26). The activity of TTLL7 is slightly reduced for tubulins from P30 mice, probably because of its broad spatial expression in the brain and the saturation of polyglutamylation by endogenous TTLL7. Given that brain tubulins from new born mice are barely glutamylated, but levels of TTLL7 transcripts are consistent during brain development, we speculate that the activity of TTLL7 may be regulated in a translational level at early stages. In contrast, the modification sites for TTLL4 to generate GT335-immunoreactive signals seem to be preserved along brain development. This could be attributed to the more specific spatial expression of TTLL4 in the brain (44), leaving the majority of the brain tubulin still available for TTLL4 modification.

Analysis of the modification sites of TTLL4 and TTLL7 revealed their incompatible site specificity in both synthetic peptides mimicking the C-terminal tails of α- and β-tubulins and the tail of a recombinant mouse tubulin. Yet, neither enzyme was convincingly evident to modify the E445 site in the α1-tail, the best identified glutamylation site in brain tubulin (45), suggesting the involvement of additional glutamylases. The incompatible site specificity of TTLL4 and TTLL7 made us wonder whether the glutamylation initiated by TTLL4 affects TTLL7 for further creation of the branch point (initiation) or elongation. We found that premodification of MTs with TTLL4 did not affect the further addition of the branch-point glutamate residues by TTLL7 but impaired its elongation activity. This is particularly interesting in light of the distinct temporal expression patterns of different TTLL polyglutamylases during brain development, making it tempting to speculate that polyglutamylated sites on tubulins may vary at different ages. Moreover, as the existing modifications could affect the efficiency of further modification at adjacent sites, the erasers assumptively come into play to ensure the removal of the obsolescent modifications and allow the right sites being modified at a certain developing stage.

The present study also identified novel glutamylation sites by TTLL4 and TTLL7 in a recombinant α1A-tubulin. These sites are located in a glutamate-rich region close to the C-terminal tail of α1A-tubulin and structurally reside on the loop between its helices H11 and H12 or the N-terminal end of the H12. This region faces to the β subunit on the outer surface of MTs (Fig. 7). The function of glutamylation at these sites remains unknown. In a cryo-EM structure of TTLL7 in complex with MT, a part of the helix α7 in TTLL7 was positioned between H11 and H12 of α-tubulin (34). In addition, a crystal structure of a kinesin–tubulin complex revealed that residues E414, E415, and E420 in α1 tubulin directly interact with the helix H4 of kinesin (46). It will be interesting to determine whether the modification of these sites affects kinesin motility or TTLL7 binding with MTs. Notably, MS/MS analysis again demonstrated the different selectivities of TTLL4 and TTLL7 for these sites, suggesting that their distinct site selectivity is rather general for their common substrates. This is possibly attributed to an intrinsic mechanism related to their structures or different catalysis machineries.

Tubulin polyglutamylation has been associated with kinesin velocity and processability (24, 47). Mutations of CCPP1, the homolog of Nna1/CCP1 in *Caenorhabditis elegans*, increased the velocity of the kinesin-2 OSM-3/KIF17 and the localization of kinesin-3 KLP-6 in sensory cilia (47). In TTLL1 mutant mice, the targeting of KIF1A, a subfamily of kinesin-3 in neuritis, was impaired, but the distribution of KIF3A (kinesin-2) and KIF5 (kinesin-1) was not altered (24). The *in vitro* system presented in this study allowed to determine how modifications by individual TTLL polyglutamylases contribute to regulation of kinesin behaviors on MTs. We showed that MTs modified by TTLL4 did not alter the affinity of kinesin-1 with MTs but moderately affected its velocity. Strikingly, kinesin-1 hardly binds to the MTs modified by TTLL7, and its dwell time on those MTs reduced to half. Binding of kinesin to MTs greatly rely on the electric interaction with the acidic-exposed residues on MTs, such as those acidic residues in the H12 of β-tubulin (46) in close proximity of its C-terminal tail. We speculate that the long glutamate side chains attached to the tubulin tails by TTLL7 might disturb the proper localization of kinesin on the shaft of MTs so that impaired its binding to MTs. Moreover, the novel region in α1A-tubulin subjected to glutamylation by TTLL4 and TTLL7 is involved in direct interaction with kinesin (46). Notably, all E414, E415, and E420, which can be modified by TTLL4, are involved in direct interaction with residues on kinesin H4 through hydrogen bonds or salt bridges. It will be interesting to explore whether the modification on E417 by TTLL7 contributes to a negative regulation of kinesin binding.

Polyglutamylation initiator TTLL4 and elongator TTLL1 contribute to the neurodegeneration in *pcd* mice (10, 11, 12). It was conceivable that this is caused by the common substrates for which TTLL4 initiates the side chain, whereas TTLL1 further elongates it. However, paradox existed as these two TTLLs were known to have different tubulin isoform preference. The present work revealed that in the tubulins from postnatal brain, a subset of α-tubulin can be modified by TTLL4, underscoring the possible common substrates of TTLL4 and TTLL1. However, further studies are needed to determine whether α-tubulin is the polyglutamylation substrate causing neurodegeneration in *pcd* mice.

## Experimental procedures

### Animals

C57BL/6J male mice of different ages were purchased from SPF Biotechnology Co Ltd and sacrificed according to a protocol of cervical dislocation (protocol number: IRM-DWLL-2021094) proved by the Institutional Animal Care and Use Committees at the Institute of Radiation Medicine, Chinese Academy of Medical Science, during day time. The brain was dissected and separated from cerebellum and snap-frozen in liquid nitrogen. The experimental procedures were approved by the institutional review board.

### Expression and purification of recombinant TTLLs

A soluble truncated version of mouse TTLL4 (NM_001014974.2), TTLL4-C639 (2) was cloned to pET28a-LIC-derived vector with an histidine–maltose-binding protein-tobacco etch virus cutting site tag at the N terminus through ligation-independent cloning. For protein expression, the plasmid was transformed into Rosetta (λDE3) and induced with 0.1 mM IPTG at 16 °C for 23 h. The harvested cells were resuspended in 10 mM Hepes (pH 7.4) containing 250 mM KCl, 25 μg/mg lysozyme, 25 μg/mg DNase, and 1 mM PMSF. The cells were disrupted by sonication on ice using an ultrasonic processor (Ningbo Scientz). The lysate was centrifuged at 40,000*g* for 40 min at 4 °C (Heraeus Multifuge X1R; Thermo Fisher Scientific). The supernatant was loaded on an equilibrated Ni-column (GE Healthcare) equipped in ÄKTA (GE Healthcare). After washing with 10 mM Hepes, pH 7.4, containing 250 mM KCl and 30 mM imidazole for three column volumes (CVs), the proteins were eluted with a linear gradient from 0 to 500 mM imidazole in 10 mM Hepes, containing 250 mM KCl. Monitored with SDS-PAGE followed by CBB staining, the fractions containing the target protein were combined and dialyzed against 10 mM Hepes, pH 7.4, containing 250 mM KCl and 0.1 mM DTT at 4 °C overnight. After concentrated using an ultrafiltration concentration tube (Millipore), the protein concentration was determined using NanoDrop (Thermo Fisher Scientific).

For TTLL7 expression and purification, mouse TTLL7 (AM690750.1) was cloned into pET28a-LIC-derived vector and expressed and purified in a similar procedure for recombinant TTLL4 as described above.

### HEK tubulin purification

HEK tubulin was purified from HEK293T cells that are cultured in suspension. Briefly, the cells were harvested, washed, and lysed by douncing on ice (20 strokes). The lysate was centrifuged at 40,000 rpm for 60 min (Beckman XPN-100 ultracentrifuge, Type 45 Ti rotor). The supernatant was collected and filtered through a 0.45 μm filter (Pall, catalog no.: 66229), and the cleared supernatant was loaded onto a TOG-based affinity purification column (48). Then, the column was successively washed with 5 CVs of BRB80, 5 CVs of BRB80 supplemented with 1 mM ATP, and 4 CVs of BRB80. Finally, tubulin was eluted with 3 CVs of BRB80 supplemented with 500 mM (NH_4_)_2_SO_4_ (Sigma–Aldrich; catalog no.: A4418). Finally, the eluate was quickly desalted into 1× BRB80 using the PD-10 column (GE; catalog no.: 17043501).

### Mouse brain tubulin purification and polymerization

Tubulins were prepared from two or three P2 (postnatal day 2/newborn), P7, P15, P30, or P60 (adult) mouse brains using an affinity column as described previously and eluted in a BRB80 buffer (48). The purity of purified tubulin was monitored using CBB staining (exemplified in Fig. S1).

### Protein electrophoresis and immunoblotting

Proteins were separated using 10% gel with an electrophoresis cells (GE Life Technologies). To separate α- and β-tubulins, 10% gel was prepared using a stock solution of 40% acrylamide solution (Meryer) supplemented with 0.54% bisacrylamide (w/v) powder (Yuanye) according to a protocol reported previously (39). After electrophoresis, proteins were transferred onto a nitrocellulose membrane using a semidry blotter (GE Healthcare). Membranes were incubated with anti-His tag (1:5000 dilution; Immuno Way), anti-glutamylation (GT335, 1:4000 dilution; Adipogen), anti-polyglutamylation (polyE, 1:4000 dilution; Adipogen), anti-α-tubulin (EP1332Y, 1:4000 dilution; Abcam or DM1A, 1:4000 dilution; Sigma), or anti-β-tubulin (anti-β-tubulin, 1:50,000 dilution; Proteintech) antibody. Immunoreactivity of proteins was visualized with WesternBright ECL (Advansta) using Western Blot Imager (Vilber) after incubation with horseradish peroxidase–labeled goat antimouse (1:5000 dilution, Bioss) or donkey anti-rabbit (1:5000 dilution, Bioss) antisera.

### Tubulin glutamylation assays of recombinant TTLLs

Glutamylation assays using porcine tubulin (Cytoskeleton) or purified mouse brain tubulin as the substrate were performed in 20 mM Hepes, pH 7.0, containing 5 mM MgCl_2_, 1 mM ATP, 1 mM glutamate, 1 mM DTT, 0.2 mg/ml tubulin, and 0.02 mg/ml denatured or intact recombinant TTLL4 or TTLL7 and incubated at 37 °C for 2 h (TTLL4) or 1 h (TTLL7) unless otherwise specified. Reactions were terminated with sample buffer and subsequently subjected to immunoblotting analysis using antibody GT335 (antibranch point glutamate), B3 (recognizing side chain with more than two glutamate residues), polyE (anti–long-chain polyglutamate), anti-β-tubulin, and anti-α-tubulin DM1A or EP1332Y. To quantify the immunoreactive signals, gray scale analysis was performed using the “Measure” tool in ImageJ (National Institutes of Health) or “Quantification” tool in Evolution-Capt Edge (Vilber) to determine the integrated densities of the bands.

### Expression analysis of TTLLs using quantitative real-time PCR

Dissected cerebellum and the rest of brain from different mice age groups (postnatal days 1, 3, 7, 15, 21, 30, or 60) were snap-frozen in liquid nitrogen, and total RNA was extracted using TRIzol reagent (CWBIO). First-strand complementary DNA was synthesized by reverse transcription using the ABScript II cDNA synthesis kit (ABclonal) according to the manufacturer's protocol. Expression analysis of TTLL4 was done with primers 5′-CTCGTCCGTTTTGCCAGTTG-3′ and 5′-TTCAATGCCCATTTGTGCCC-3′. Primers 5′-TTTGACGTGCGCCTCTATGT-3′ and 5′-CAGGTGCATGAACTGGTTGC-3′ were used for expression analysis of TTLL5. Primers 5′-ATGAAC GGTTTGAGCGCAATG-3′ and 5′-ACGTGAGGTTCTGCCACAAT-3′ were used to evaluate TTLL7 expression. As a loading control, β-actin was amplified using primers 5′-GGCTGTATTCCCCTCCATCG-3′ and 5′-CCAGTTGGTAACAATGCCATGT-3′. The RNA level was quantified using the standard curve method and normalized to β-actin level of individual samples. Three mice were included in each age group. The values of TTLL expression normalized to β-actin were represented as mean ± SEM.

### Glutamylation assay of recombinant TTLLs for peptides

A synthetic peptide corresponding to the C terminus of detyrosinated α1A/B-tubulin (VGVDSVEGEGEEEGEE, peptide A) or β2-tubulin (DEQGEFEEEEGEDEA, peptide B; SciLight Biotechnology) was incubated with the denatured or intact recombinant TTLL4 or TTLL7 (0.02 mg/ml) for overnight at 18 °C at a 10:1 ratio of peptide to enzyme in 20 mM Hepes (pH 7.0), 5 mM MgCl_2_, 1 mM DTT, 1 mM ATP, and 1 mM [^13^C5]-l-glutamate (Zhongkehengyi). Reactions were terminated by addition of an equal volume of 10% TFA and dried with a speed-vacuum (Thermo Fisher Scientific). Samples were then desalted using zip-tip (Millipore), eluted with 50% acetonitrile (ACN) containing 0.1% TFA, and then subjected to LC–MS analysis.

### LC–MS/MS analyses of glutamylated peptides

Peptides from the reactions were analyzed by a Fusion Lumos mass spectrometer coupled with an Easy-nLC 1200 system (Thermo Fisher Scientific). Briefly, peptides were loaded onto a 150 μm × 2 cm self-packed C18 trap column (particle size 3 μm; Dr MASCH GmbH) and separated on a 150 μm × 30 cm self-packed C18 analytical column (particle size 1.9 μm; Dr MASCH GmbH). Mobile phase A consisted of 0.1% formic acid (FA), and mobile phase B consisted of 80% ACN and 0.1% FA. Gradients were run as follows: 4 to 8% B for 2 min, 8 to 28% for 43 min, 28 to 36% for 8 min, 36 to 100% for 2 min, and 100% for 5 min. The mass scan range was set from 250 to 1500 *m/z*, and the full MS resolution was set as 120,000. The automated gain control (AGC) target was set as 4E5, and the maximum injection time was set as 50 ms. The mass spectrometer was operated in the data-dependent mode with ion trap scan rate “Rapid” selected for higher-energy collision dissociation fragmentation with 30% normalized collision energy. The isolation window for MS/MS was set as 1.6 *m/z*. The MS/MS spectra were acquired with 5E4 AGC and 30 ms maximum injection time. The mass tolerance for precursor ions was 10 ppm, and the mass tolerance for fragment ions was 10 ppm. The false discovery rate was set to 1% for peptide identification. The conversion rate was calculated based on the area of peaks of the substrate and +1E product of corresponding *m/z*. The ions for substrates or products with corresponding theoretical *m/z* (±10 ppm) were searched against all spectra, and the area of peaks was integrated manually.

### Mass spectrometric analysis for glutamylation on recombinant tubulins

The bands of tubulins in CBB-stained hydrogel after SDS-PAGE were excised and subjected to in-gel digestion as described previously (49). Peptides were analyzed by a Fusion Lumos mass spectrometer accompanied with an Easy-nLC 1200 system. Peptides were loaded onto a 150 μm × 2 cm self-packed C18 trap column and separated on a 150 μm × 30 cm self-packed C18 analytical column with a gradient of 5 to 35% phase B solution (containing 0.1% FA and 80% ACN) over 120 min. The mass scan range was set from 350 to 1500 *m/z*, and the full MS resolution was set as 120,000. The AGC target was set as 5E5, and the maximum injection time was set as 50 ms. The mass spectrometer was operated in the data-dependent mode with ion trap scan rate “Rapid” selected for higher-energy collision dissociation fragmentation with 35% normalized collision energy. The isolation window for MS/MS was set as 1.6 *m/z*. The MS/MS spectra were acquired with 100,000 AGC and 50 ms maximum injection time. All raw files were analyzed with a Proteome Discoverer software (version 2.2, Thermo Fisher Scientific). Peak lists were searched against the UniProt protein database and sequence of α1A/β2A tubulins. Search parameters included a minimum peptide length of six, two missed cleavage sites, protease specificity with trypsin, ^13^C5-labeled-glutamylation fixed modification, and oxidized methionine variable modification. The mass tolerance for precursor ions was 10 ppm, and the mass tolerance for fragment ions was 7 ppm. The false discovery rate was set to 1% for peptide and protein identification.

### Kif5c-GFP purification

The plasmid of Kif5c-GFP (amino acids: 1–430) was a gift to Xin Liang form Jonathon Howard (Yale University), and this construct was originally from Rob Cross. Kif5c-GFP was expressed in the *BL21 Escherichia coli* strain and lysed using sonication in the lysis buffer (50 mM NaPO_4_ buffer [pH 7.5], 300 mM NaCl, 0.1 mM ATP, 10% glycerol, 10 mM imidazole, 1 mM DTT, and 0.1% Tween-20). The lysate was centrifuged at 40,000 rpm for 60 min at 4 °C. The supernatant was filtered through a 0.45 μm filter and loaded onto a Ni-sepharose column. After washing with the washing buffer (50 mM NaPO_4_ buffer [pH 7.5], 300 mM NaCl, 0.1 mM ATP, 10% glycerol, 10 mM imidazole, and 1 mM DTT), the protein was eluted using the washing buffer supplemented with a continuous imidazole gradient (20–300 mM). The fractions with best purity were pooled together and then desalted into the storage buffer (BRB80, 100 mM KCl, 0.1 mM ATP, 10% glycerol, and 1 mM DTT; BRB80: 80 mM Pipes/KOH [pH 6.9], 1 mM MgCl_2_, and 1 mM EGTA) using the PD-10 desalting column (GE; catalog no.: 17085101). The final purified protein was frozen in liquid nitrogen and stored at −80 °C.

### MT preparation and modification and the binding and motility assay of Kif5c

GMPCPP-MTs were polymerized using a mixture of 20 μM tubulin extracted from mouse brain, 1 mM GMPCPP (Jena Bioscience; catalog no.: NU-405L) and 4 mM MgCl_2_ in BRB80, which was incubated for 2 h at 37 ˚C. Polymerized MTs were collected using an air-driven ultracentrifuge (Beckman; catalog no.: 340401), which were then resuspended in BRB80 with 20 μM taxol (Cell Signaling Technology; catalog no.: 9807), and stored at 37 ˚C. Part of MTs was treated with TTLL4 or TTLL7 for 2 h at room temperature of 37 °C. Then, MTs were immobilized on the surface of a cover glass using the tubulin antibody links (Sigma; catalog no.: T7816). Next, Kif5c-GFP was added into the flow cell in the imaging buffer (BRB80 supplemented with 1 mM ATP, 20 μM taxol, 80 mM d-glucose, 0.4 mg/ml glucose oxidase, 0.2 mg/ml catalase, 0.8 mg/ml casein, 1% β-mercaptoethanol, and 0.001% Tween-20). Images were recorded every 100 ms with a 50 ms exposure. The sample was kept at 35 °C using a temperature controller (Tokai Hit). Images were recorded using a TIRFM (Olympus).

### Analysis of the dwell time of kinesin

The dwell time was defined as the duration of a kinesin-1 molecule making processive movement along MTs, and we used kymographs to measure the dwell time of individual kinesin-1 molecules. Note that the binding events whose duration was shorter than 0.2 s were not included in our dataset. The statistical distribution of dwell time (from three experiments) was fitted to a single exponential function.

### Statistical methods

Student’s *t* test and Mann–Whitney *U* test were used to compare independent samples for statistical significance. Significance was set at *p* < 0.05. Student’s *t* test was performed using Microsoft Excel. Mann–Whitney *U* test was performed by an online Mann–Whitney *U* test calculator (socscistatistics.com).

## Data availability

All datasets are included in this article.

## Supporting information

This article contains supporting information.

## Conflict of interest

The authors declare that they have no conflicts of interest with the contents of this article.
