# Supplementary material for: The distinct initiation sites and processing activities of TTLL4 and TTLL7 in glutamylation of brain tubulin
Source: J Biol Chem. 2023 Jun 14;299(7):104923. doi: 10.1016/j.jbc.2023.104923 (PMC10404701; doi:10.1016/j.jbc.2023.104923)
Supplement: Supporting Figures S1–S5 [file mmc1.docx]

**Supporting information**

**The distinct initiation sites and processing activities of TTLL4 and TTLL7 in glutamylation of brain tubulin**

Xinyue Zhang^1#^, Xiangxiao Li^1#^, Wei Chen^2^, Yujuan Wang^1^, Lei Diao^3^, Yan Gao^1^, Heyi Wang^1^, Lan Bao^3^, Xin Liang^2^, and Hui-Yuan Wu^1^*

^1^School of Pharmaceutical Science and Technology, Tianjin University, Tianjin, 300072, China

^2^ IDG/McGovern Institute for Brain Research, School of Life Sciences, Tsinghua University, Beijing 100084, China

^3^ State Key Laboratory of Cell Biology, Shanghai Institute of Biochemistry and Cell Biology, Center for Excellence in Molecular Cell Science, Chinese Academy of Sciences, Shanghai 200031, China

**Running title:** TTLL4 and TTLL7 modify different sites on tubulins

# Equal contribution

* Corresponding Author

**Supporting information list:**

Figure S1

Figure S2

Figure S3

Figure S4

Figure S5


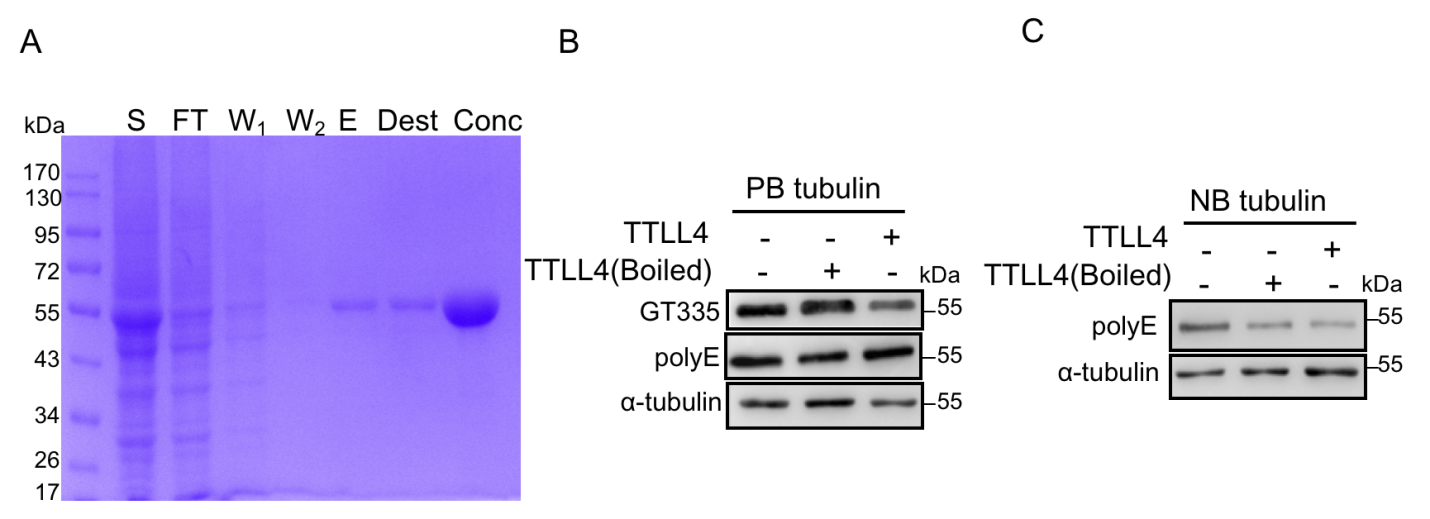


**Fig. S1 The TTLL4 activity can be masked with tubulin of some resources as the substrate.** (A) Representative image of CBB staining of fractions during brain tubulin purification. S, supernatant; FT, flowthrough; W1, 1^st^ wash; W2, 2^nd^ wash; E, eluate; Dest, desalted; Conc, Concentrated; (B) The baseline glutamylation signals of porcine brain (PB) tubulin overwhelmed the increase in glutamylation by recombinant TTLL4. (C) TTLL4 did not alter the polyE signals in newborn mouse brain (NB) tubulin.


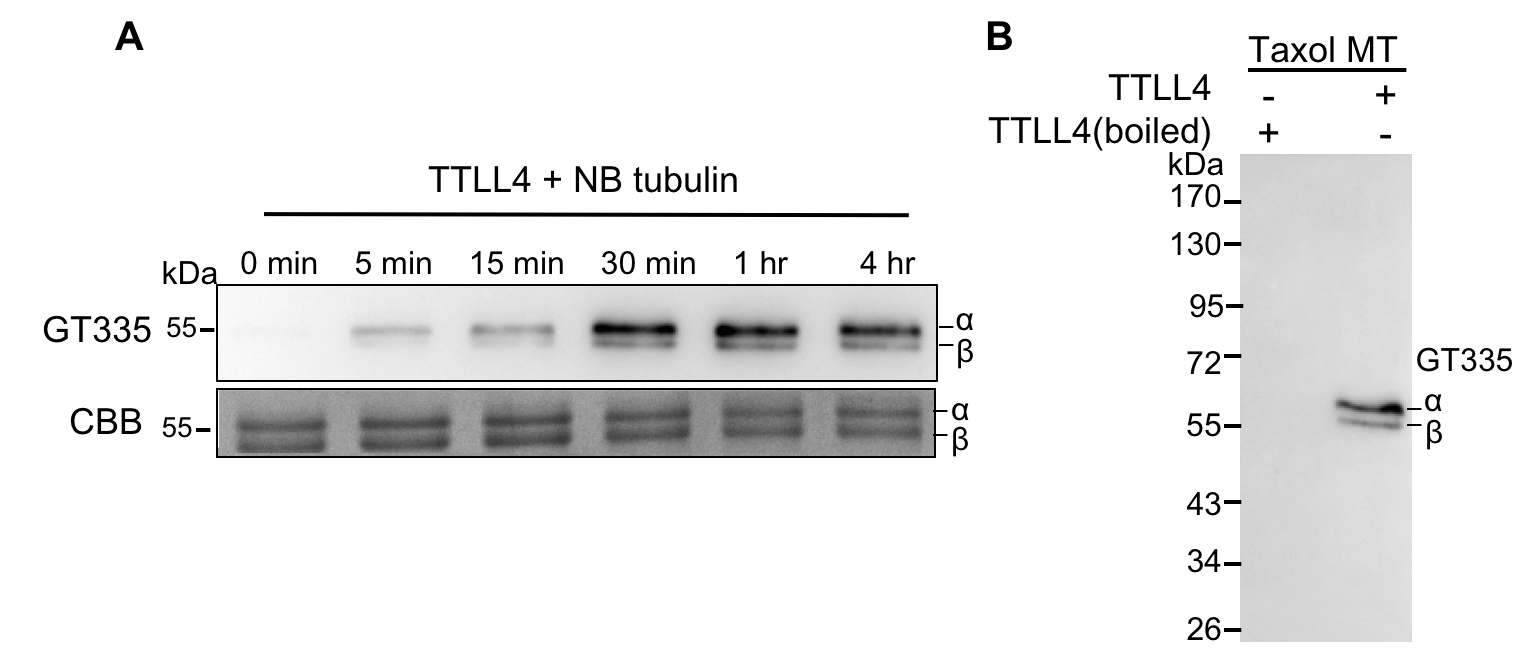


**Fig. S2 Validation of the preference of recombinant TTLL4 for the α-isoform of MT from newborn mouse brain using different preparation.** (A) The time course of modification of α- and β-isoforms of NB mouse brain tubulin by the recombinant TTLL4. GT335-reactivity of α-isoform kept being higher than that of β-isoform during incubation. CBB-stained gel demonstrated the presence of comparable amount of both isoforms of tubulin in the reactions. (B) With microtubule (MT) purified from newborn mouse brain using an irreversible polymerization method (Vallee R.B. (1986) *Methods Enzymol*, **134**) as the substrate, the recombinant TTLL4 also exhibits a higher activity for α-tubulin.


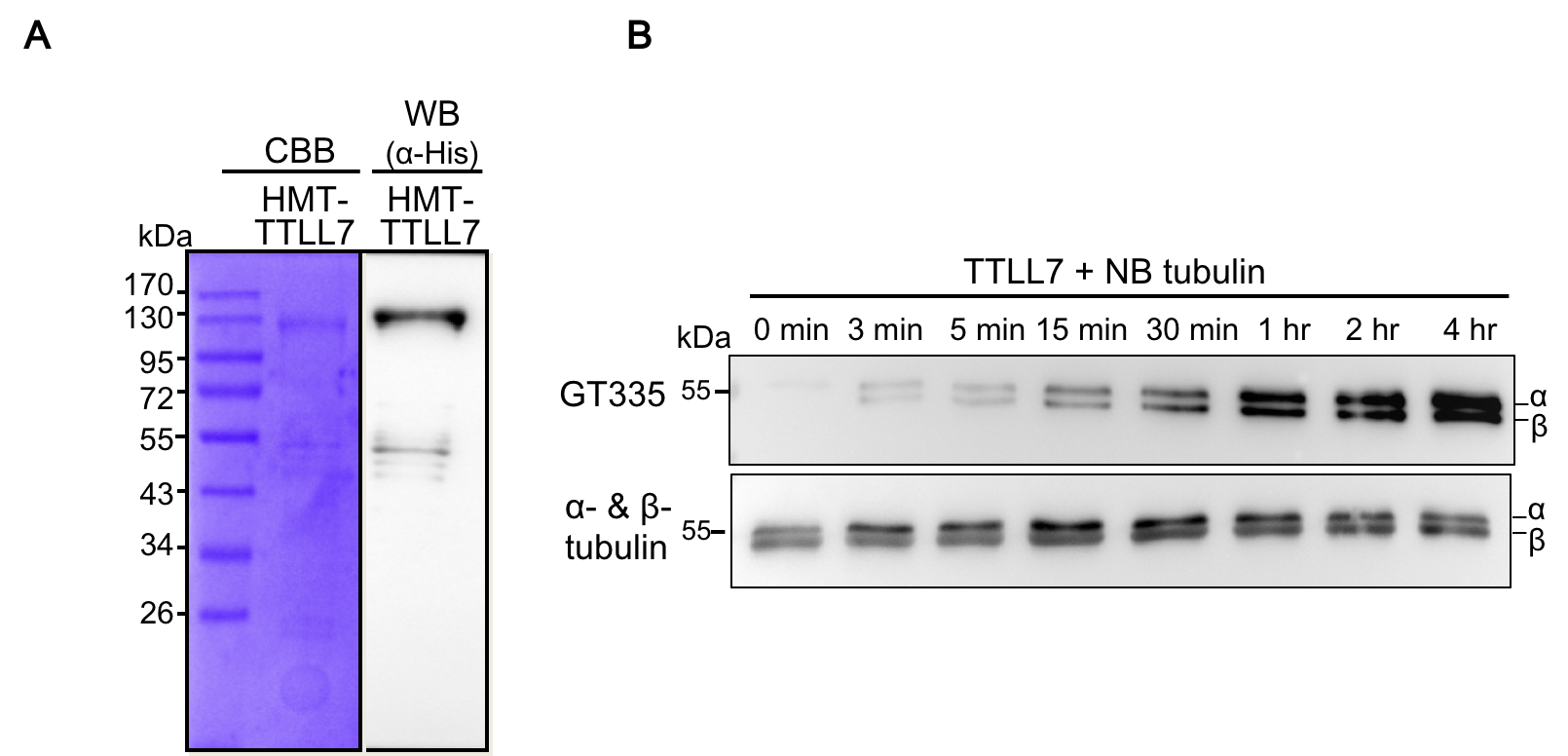


**Fig. S3 Recombinant TTLL7 produced comparable GT335-reactive isoforms for newborn mouse brain tubulin.** (A) CBB staining shows the major band of purified protein of predicted molecular weight (left panel), which is immunoreactive for tan his-tag antibody. (B) The time course of modification of α- and β-isoforms of NB mouse brain tubulin by the recombinant TTLL7. GT335-reactivity is comparable between α- and β-isoforms during incubation.


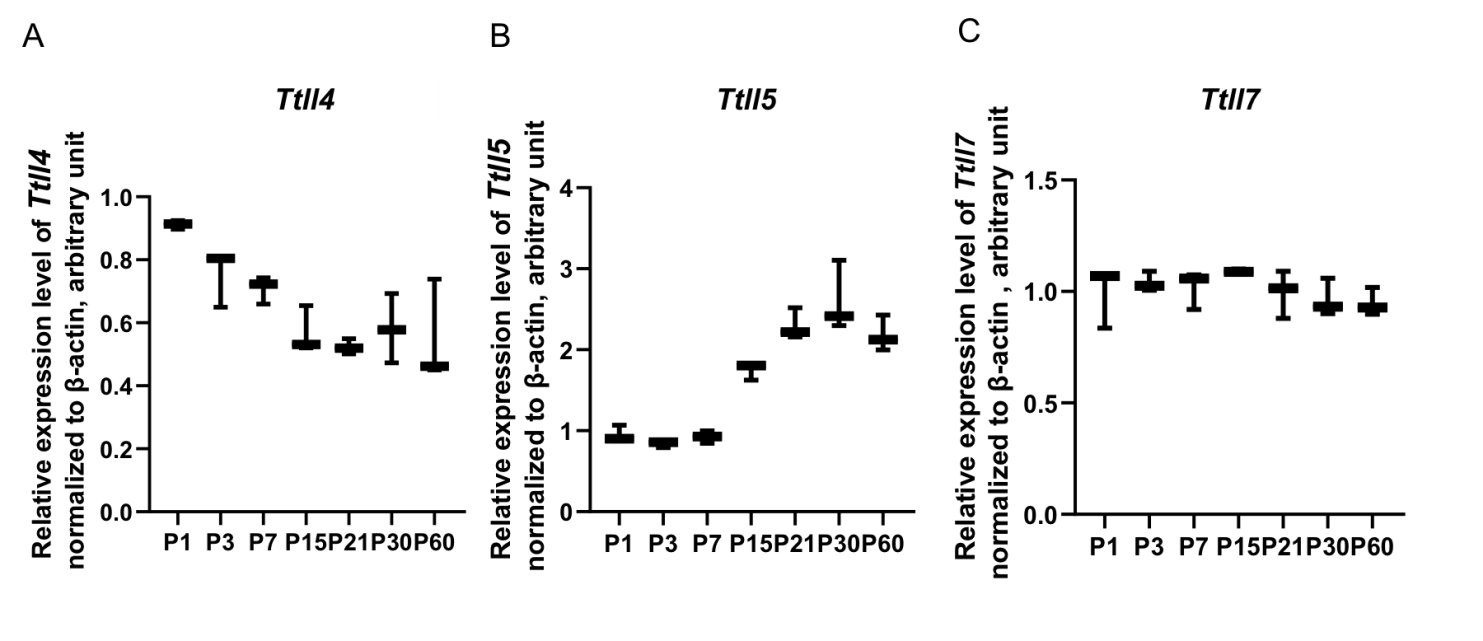


**Fig. S4 Quantitative real-time PCR detection of TTLL4, TTLL5, and TTLL7 expression in developing mouse brain** Total RNA was prepared from the brain of P1, P3, P15, P21, P30, and P60 mice. Relative mRNA quantity was normalized to housekeeping gene *β*-actin. Bars represent mean ± s.e.m. (error bars) of determinations of three individuals. Temporal expression of (A) TTLL4, (B) TTLL5, and (C) TTLL7 during brain development. (A) The level of TTLL4 transcripts is high at the birth and slightly reduced during further development. (B) TTLL5 expression is low in the first week after birth and increased during further development, whereas (C) the expression of TTLL7 keeps consistent in developing brain.

**
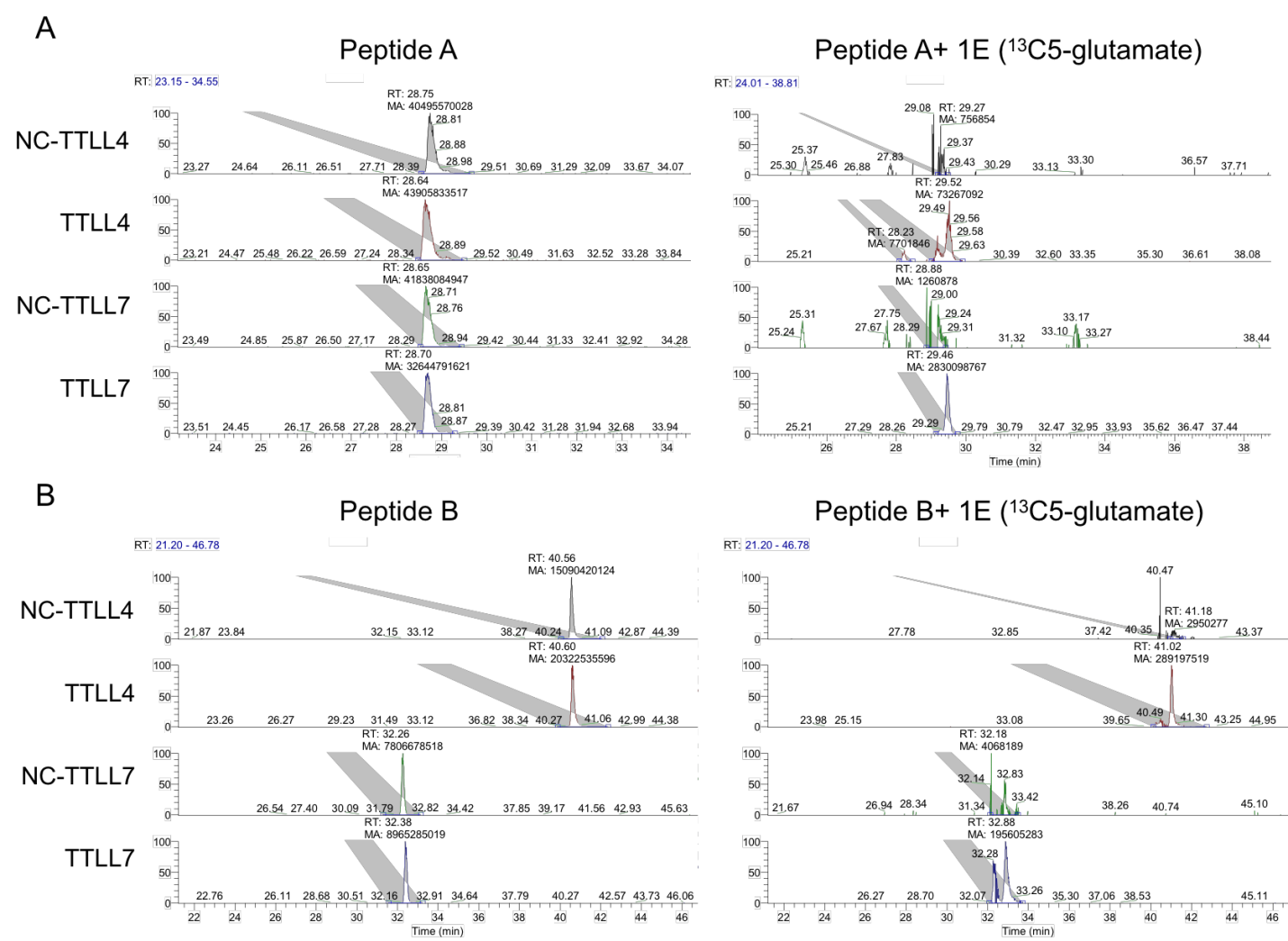
**

**Fig. S5 Examples of chromatograph used for quantification of the conversion rate of peptide A or peptide B to generate +1E products by TTLL4 or TTLL7. (A)** LC-MS analysis for the activity of TTLL4 and TTLL7 with α1A-tubulin tail peptide (VGVDSVEGEGEEEGEE, peptide A) as the substrate to generate +1E products. Ions corresponding to the m/z of the substrate (825.3284-825.3450, left panel) or the product (892.3572-892.3751, right panel) with 10 ppm tolerance were extracted and the areas of peaks were manually integrated (MA). (B) LC-MS analysis for the activity of TTLL4 and TTLL7 with β2-tubulin tail peptide (DEQGEFEEEEGEDEA, peptide B) as the substrate to generate +1E products. Ions corresponding to the m/z of the substrate (871.3047-871.3221, left panel) or the product (938.3335-938.3523, right panel) with 10 ppm tolerance were extracted and the areas of peaks were manually integrated. The conversion rate was estimated according to MA_product_/(MA_substrate_+MA_product_)*100%.
